# Distant origin of glioblastoma recurrence: neural stem cells in the subventricular zone serve as a source of tumor reconstruction after primary resection

**DOI:** 10.1186/s12943-025-02273-2

**Published:** 2025-03-04

**Authors:** Xue Li, Hyun Jung Kim, Jihwan Yoo, Yeonhee Lee, Chang Hyun Nam, Jonghan Park, Soon-Tae Lee, Tae Min Kim, Seung Hong Choi, Jae-Kyung Won, Sung‑Hye Park, Young Seok Ju, Jong Bae Park, Se Hoon Kim, Jong Hee Chang, Hong-Gyun Wu, Chul-Kee Park, Jeong Ho Lee, Seok-Gu Kang, Joo Ho Lee

**Affiliations:** 1 Department of Radiation Oncology, Seoul National University College of Medicine, Seoul National University Hospital, Seoul, South Korea; 2 Cancer Research Institute, Seoul National University College of Medicine, Seoul, South Korea; 3 Institute of Radiation Medicine, Medical Research Center, Seoul National University College of Medicine, Seoul, South Korea; 4 Department of Radiation Oncology, The Affiliated Cancer Hospital of Zhengzhou University & Henan Cancer Hospital, Zhengzhou, People’s Republic of China; 5 Graduate School of Medical Science and Engineering, Korea Advanced Institute of Science and Technology (KAIST), Daejeon, South Korea; 6 Department of Anatomy, Korea University College of Medicine, Seoul, South Korea; 7 Department of Neurosurgery, Brain Tumor Center, Gangnam Severance Hospital, Yonsei University College of Medicine, Seoul, South Korea; 8 Department of Neurology, Seoul National University College of Medicine, Seoul National University Hospital, Seoul, South Korea; 9 Department of Internal Medicine, Seoul National University College of Medicine, Seoul National University Hospital, Seoul, South Korea; 10 Department of Radiology, Seoul National University College of Medicine, Seoul National University Hospital, Seoul, South Korea; 11 Department of Pathology, Seoul National University College of Medicine, Seoul, South Korea; 12 Department of Cancer Biomedical Science, Graduate School of Cancer Science and Policy, National Cancer Center, Goyang-si, Korea; 13 Department of Pathology, Severance Hospital, Yonsei University College of Medicine, Seoul, South Korea; 14 Department of Neurosurgery, Brain Tumor Center, Severance Hospital, Yonsei University College of Medicine, Seoul, South Korea; 15 Department of Neurosurgery, Seoul National University College of Medicine, Seoul National University Hospital, Seoul, South Korea; 16 Genomic Medicine Institute, Medical Research Center, Seoul National University, Seoul, South Korea; 17 Sovargen Inc, Daejeon, South Korea; 18 Department of Medical Sciences, Yonsei University Graduate School, Seoul, South Korea

**Keywords:** Glioblastoma, Recurrence, Subventricular zone, Neural stem cells

## Abstract

Glioblastoma (GBM) is the most aggressive and common type of primary malignant brain cancer in adults. GBM often recurs locally near the resection cavity (RC) following the surgical removal of primary tumors. Recent research has reported that neural stem cells (NSCs) in the subventricular zone (SVZ) harboring cancer-driving mutations serve as the cells of origin for human GBM. However, the pathological role of tumor-initiating NSCs in the SVZ in tumor recurrence remains to be elucidated. Here, we explore the potential contribution of mutation-harboring NSCs in the SVZ to tumor recurrence around the RC following surgical resection. Our hypothesis emerged from performing deep sequencing of longitudinal tissues from 10 patients with GBM, including (i) tumor-free SVZ tissue, (ii) primary tumor tissue, (iii) recurrent tumor tissue, and (iv) blood. As a result of this sequencing, we observed evidence suggesting that recurrent tumors show genetic links to the SVZ in 60% (6/10) of patients, which are distinct from the primary tumors. Using a genome-edited mouse model, we further identified that mutation-harboring NSCs appeared to migrate to the RC through the aberrant growth of oligodendrocyte progenitor cells, potentially contributing to the reconstruction of high-grade malignant gliomas in the RC. This process was associated with the CXCR4/CXCL12 axis, as supported by RNA sequencing data from human recurrent GBM. Taken together, our findings suggest that NSCs in human SVZ tissue may play a role in GBM recurrence, potentially highlighting a novel distant contributor of recurrence.

**Supplementary Information:**

The online version contains supplementary material available at 10.1186/s12943-025-02273-2.

## Introduction

Glioblastoma (GBM) is the most aggressive diffuse glioma, and it accounts for 54% of all gliomas and 16% of all primary brain tumors [1, 2]. The current standard of care for newly diagnosed GBM is maximal surgical resection followed by concurrent radiotherapy and temozolomide [3, 4]. Although there have been advancements in diagnosis and treatment, the prognosis of patients with GBM has not significantly improved, as it has a median survival of 14–20 months [5, 6]. The poor prognosis of GBM can be attributed to several interrelated factors, including infiltrative growth, complex genetic and molecular heterogeneity, therapy resistance, and a pro-tumorigenic microenvironment, all of which contribute to a high tumor recurrence rate nearing 100% [7, 8]. Recurrence has been shown to occur in approximately 80% of aggressively treated patients within the resection cavity (RC), even after gross total resection of the primary tumor [9, 10]. Thus, we hypothesize that alternative cellular origins, in addition to residual tumor cells at the resection margin, may be responsible for tumor recurrence.

Recent studies have shown that neural stem cells (NSCs) in the subventricular zone (SVZ) harbor cancer-driving mutations and potentially serving as the cellular origin for human GBM [11–15]. These cells are hypothesized to play a role in tumor recurrence due to their retained tumor-initiating capacity and migratory potential [16]. Adult NSCs in the SVZ typically migrate along the rostral migratory stream to the olfactory bulb [17]; however, these cells can change their path to move toward damaged areas following brain injury [18]. This altered migration is guided by chemokines and cytokines that are released from the injured brain region, including tumor necrosis factor-alpha (TNFα), interferon-gamma (IFNγ), monocyte chemotactic protein-1 (MCP-1), and stromal-derived-factor-1 (SDF-1) [19–21]. Although this migration diminishes with age [22, 23], adult NSCs still possess the ability to migrate and differentiate following ischemic injury [24, 25]. However, the specific association between mutation-harboring NSCs in SVZ and GBM recurrence after maximal surgical resection remains poorly understood. To address this, we established a genome-edited mouse model by labeling mutation-harboring SVZ NSCs with a different reporter protein than that used for tumor cells, thus allowing us to trace the origin of GBM recurrence. We also conducted deep sequencing of longitudinal tissues from 10 GBM patients, with the results providing evidence indicating that mutation-harboring NSCs in SVZ are involved in the process of tumor reformation within the RC following complete surgical excision of the primary tumor.

## Results

### Genetic profiles and clonal relationship linking the SVZ with primary and recurrent tumors

**Fig. 1:**
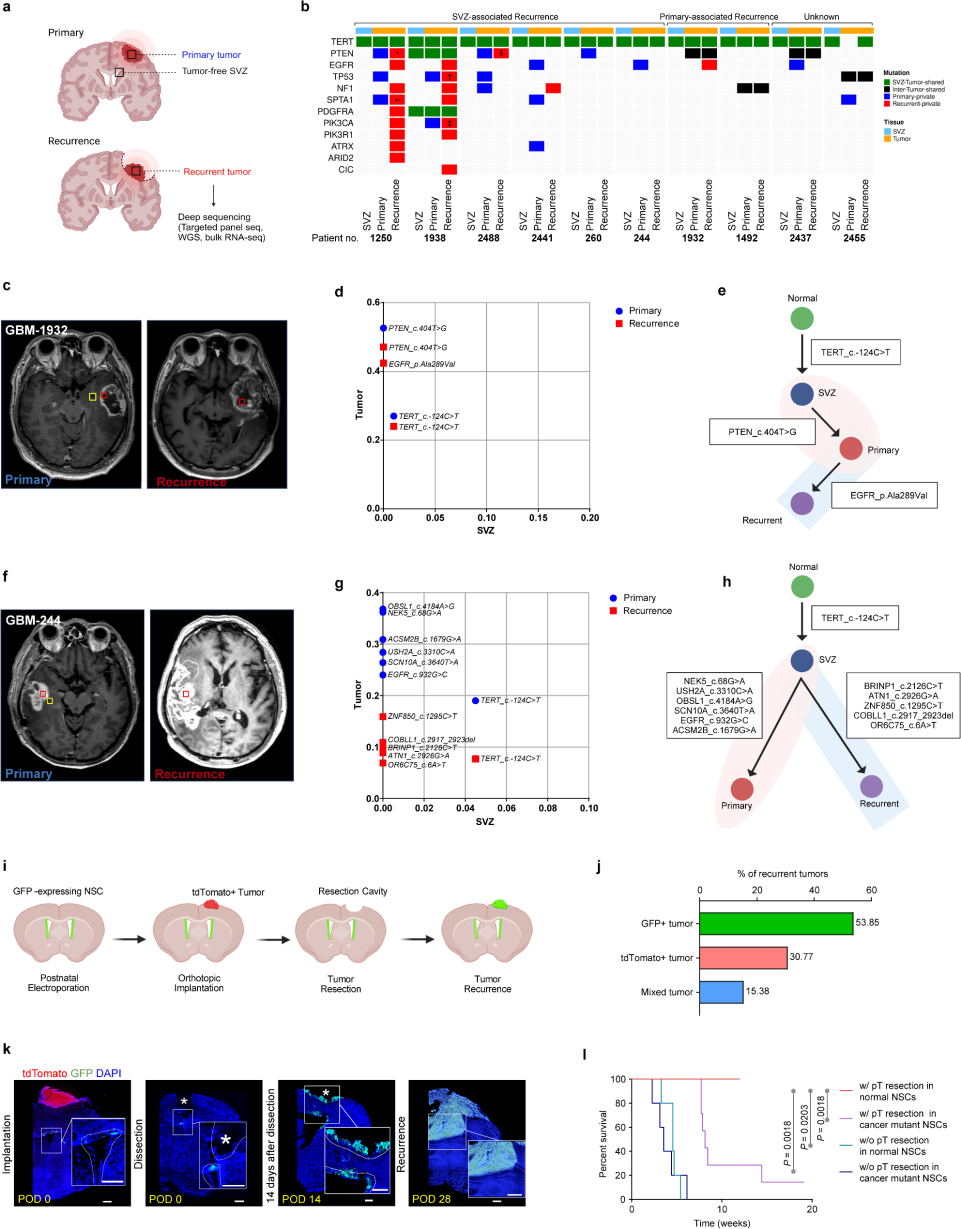
Evidence of SVZ NSC involvement in glioblastoma recurrence with cancer-driving mutations. **a**, Schematic diagram of sampling for human longitudinal paired primary, recurrent tumors and SVZ tissues, which was followed by application for deep sequencing. Created with Biorender.com. **b**, Oncoplot displaying DNA panel sequencing results from longitudinal paired primary and recurrent tumors and SVZ tissues from 10 patients. Mutations are categorized as SVZ-Tumor-shared (green), Inter-Tumor-shared (black), Primary-private (blue), and Recurrent-private (red). An asterisk (*) denotes a distinct PTEN mutation present in the recurrent tumor of GBM-1250 (g.87933010 C > G) compared to the primary tumor (p.Asp252Val). Double asterisks (**) indicate a distinct SPTA1 mutation present in the recurrent tumor of GBM-1250 (g.158680532 C > T) compared to the primary tumor (g.158666421 A > C). A dagger (†) marks a TP53 mutation found in the recurrent tumor of GBM-1938 (c.524 C > T) compared to the primary tumor (c.613 A > G). A double dagger (‡) marks PIK3CA mutations found in the recurrent tumor of GBM-1938 (p.Gly118Asp) compared to the primary tumor (g.179239627 A > T). A section sign (§) highlights a distinct PTEN mutation in the recurrent tumor of GBM-2488 (g.87864488-87864493del) compared to the primary tumor (g.87961042_87961045del). Annotation bars indicate tissue type and recurrence pattern, classified as SVZ-associated, Primary-associated, or Unknown. **c**, MRI image of the sampling sites in GBM-1932 patient, with the red box representing the paired tumors and the yellow box representing the SVZ. **d**, VAF scatterplot of mutations from triple-matched samples of GBM-1932. Each point represents an individual somatic mutation identified in the samples. Mutations that are private to the SVZ (x-axis), private to tumor (y-axis), or shared between the two tissues are plotted according to their VAF. Blue dots indicate mutations found in the primary tumor, while red dots represent mutations in the recurrent tumors. **e**, Phylogenetic evolutionary tree of the evolutionary process of primary and recurrence in patient GBM-1932. Created with Biorender.com. **f**, MRI image of the sampling sites in GBM-244 patient, with the red box representing the paired tumors and the yellow box representing the SVZ. **g**, VAF scatterplot of mutations from triple-matched samples of GBM-244. Each point represents an individual somatic mutation that is identified in the samples. Mutations that are private to the SVZ (x-axis), private to tumor (y-axis), or shared between the two tissues are plotted according to their VAF. Blue dots indicate mutations found in the primary tumor, while red dots represent mutations in the recurrent tumors. **h**, Phylogenetic evolutionary tree of the evolutionary process of primary and recurrence in patient GBM-244. **i**, Schematic diagram of transplanted primary tumor removal model. First, primary glioblastoma cells were dissected from the brain of mice bearing tdTomato-positive primary tumor and cultured as suspension cells. Four weeks after electroporation, these cells were implanted into the cerebral cortex of mice with GFP-positive SVZ NSCs. One week later, a bulky tumor formed and was surgically removed. Recurrent tumors were shown in the RC post-resection. Created with Biorender.com. **j**, Bar chart showing the proportion of recurrent tumors in GFP, tdTomato, and Mix (mixture of GFP and tdTomato). **k**, Representative coronal sections of mice stained with DAPI showing sections from tumor-implanted mice at seven days after implantation as well as 0, 14, and 28 days after surgical removal of the primary tumor. The asterisk indicates the RC. The white dashed line indicates the SVZ and RC sites. Scale bars, 500 μm. **l**, Kaplan-Meier survival analysis of mice (*n* = 5 per group, log-rank test) in the control group and SVZ-mutated group with or without primary tumor resection

To investigate whether SVZ NSCs are clonally connected to primary and recurrent tumors, we analyzed the mutation patterns in paired primary and recurrent tumor tissues, along with tumor-free SVZ samples. Recurrence samples were prospectively collected between 2013 and 2022 from patients exhibiting features of clonal evolution at the time of their primary tumors after gross total resection, defined by (i) low-frequency cancer-driving mutations in the SVZ, and (ii) the emergence of tumor-private mutations in the primary tumor. We initially performed deep sequencing on three matched tissue samples from each patient with IDH-wildtype GBM, including (i) tumor-free SVZ tissue, (ii) primary tumor tissue, and (iii) blood. Deep hybrid capture sequencing (median depth: 1063x) was conducted to detect somatic base substitution and short indels in genes commonly mutated in GBM. From this initial cohort, 10 patients who experienced tumor recurrence after gross total resection of the primary tumors were selected for further analysis through deep sequencing of their recurrent tumor samples (Fig. 1a).

In the 10 patients with recurrence samples, *TERT* promoter mutations were consistently shared between the tumors and their paired SVZ, indicating a clonal lineage link (Fig. 1b). Additionally, two patients (20%)―Patient IDs 1932 and 1492―exhibited additional mutations shared between the primary and recurrent tumors that were distinct from those found in the SVZ (Fig. 1c-e, Supplementary Fig. 1). We categorized this recurrence pattern as “primary-associated,” as the recurrent tumors appeared to evolve from dominant clones of the primary tumor within the same clonal lineage, suggesting local recurrence likely driven by residual infiltrative tumor cells near the RC. For two other patients—Patient IDs 2455 and 2437—we were unable to determine the clonal lineage of the recurrence and, therefore, categorized them as “unknown.”

In contrast, the other six patients (60%)―Patient IDs 1250, 1938, 2488, 2441, 260, and 244―did not exhibit any inter-tumor shared mutation between the primary and recurrent tumors excepted the mutations shared between SVZ and tumors (Fig. 1b, Supplementary Fig. 2). *TERT* promoter mutations were consistently shared among the SVZ, primary, and recurrent tumors in all cases. Notably, GBM-1938 shared three mutations, including *TERT* promoter, *PDGFRA*, and *PTEN* (Supplementary Fig. 2d-f). We categorized this recurrence pattern as “SVZ-associated,” as the high-allele frequency, tumor-private mutations of the primary tumors (primary-private mutations) became extinct in the recurrent tumors in these patients, indicating a lack of direct genetic linkage between the primary and recurrent tumors. Additionally, recurrent tumors exhibited de novo genetic variations (recurrent-private mutations) or reversion to normal alleles due to the loss of primary-private mutations. These findings suggest that the recurrent tumors may undergo independent clonal evolution and maintain genetic links to the SVZ, distinct from the primary tumor, especially following the complete resection of the primary tumors. To further investigate the dynamics of primary-private and/or recurrent-private somatic mutations, we performed whole genome sequencing (WGS) followed by deep amplicon sequencing for validation in a single patient who harbored the *TERT* promoter mutation (C228T) shared among the SVZ, primary, and recurrent tumors (Fig. 1f). In this case, we identified 10 passenger mutations in addition to the *EGFR* mutation. Notably, five primary-private mutations were absent in the recurrent tumor, while five recurrent-private mutations were not present in the primary tumor (Fig. 1g, h). Additionally, the copy number variation profile shifted from the primary to the recurrent tumors (Supplementary Fig. 3).

As a result, 60% of patients demonstrated no direct clonal linkage between primary and recurrent tumors, except for the shared mutations involving the SVZ. The distinct mutational patterns observed in recurrent tumors suggest that recurrence may originate from early progenitor clones in tumor evolution—harboring only shared mutations—rather than evolving directly from the dominant primary tumor clone. This model, in which recurrence emerges through a branched evolutionary trajectory from an early progenitor, aligns with previous studies comparing primary and recurrent tumors [26–28]. Notably, the genetic profiles of the SVZ, characterized by the presence of shared mutations without tumor-private mutations, suggest that the SVZ may serve as such an early progenitor driving recurrence independently of the primary tumor. Alternatively, other early progenitor clones responsible for recurrence may have remained undetected in primary tumor sequencing due to spatial heterogeneity and the effects of treatment on clonal distribution in GBM. Given the limitations of cross-sectional human genomic data in resolving these hypotheses, we propose in vivo modeling and time-sequenced longitudinal observations to investigate whether the SVZ has the potential to drive independent tumor recurrence.

### Multiple cellular origins for tumor recurrence

To test the hypothesis that GBM recurrence directly originates from NSCs in the SVZ, we developed a mouse model of tumor recurrence (Fig. 1i). First, we used postnatal electroporation to generate primary tumors by introducing *Trp53*, *Pten* and *Egfr* mutations into NSCs of the SVZ [12]. This single vector expressed Cre recombinase along with highly efficient single-guide RNAs (sgRNAs) targeting *Trp53* and *Pten* in the lateral ventricle of LSL-EGFRviii; LSL-Cas9-GFP mice (Supplementary Fig. 4a-c). At four weeks of age, we transplanted tdTomato-expressing mouse GBM cells into the cerebral cortex. To determine the origin of tumor recurrence, we isolated tumor cells from the same mouse model, which had been labeled with a different reporter protein and cultured them in vitro as tumorspheres prior to transplantation. These cells formed a solid tumor mass within one week, which we then removed through maximal surgical resection. Recurrent tumors were observed four weeks post-surgery, where the color of fluorescent proteins in tumor cells indicated the cellular origin of recurrent tumor.

Interestingly, all observed recurrent tumors developed within the RC, with 53.9% of recurrent tumors (7 out of 13 cases) being GFP-positive. This indicates that GFP-labeled SVZ cells had migrated and reconstructed secondary tumors, which was identified as recurrence within the RC according to the clinical timeline (Fig. 1j, Supplementary Fig. 7b). Migration of GFP-positive SVZ cells to the RC was observed even two weeks post-surgery (Fig. 1k). Despite the extensive resection, residual tdTomato-labeled tumor cells repopulated at the surgical margin, thus generating tdTomato-positive tumors (30.8%), ultimately suggesting direct recurrence from primary residual tumor cells. The remaining 15.4% tumors comprised both cell types, suggesting a mixture of both origins. Survival analysis for our mouse model for GBM recurrence revealed that recurrent tumors originating from mutation-harboring SVZ cells were associated with the worst prognosis, with a median survival time of approximately 8 weeks, when compared to recurrent tumors derived from residual primary tumors (Fig. 1l). These findings prompted further exploration of the possible mechanisms underlying GBM recurrence originating from mutation-harboring SVZ NSCs.

### Mouse modelling of post-resection tumor development driven by NSCs

**Fig. 2:**
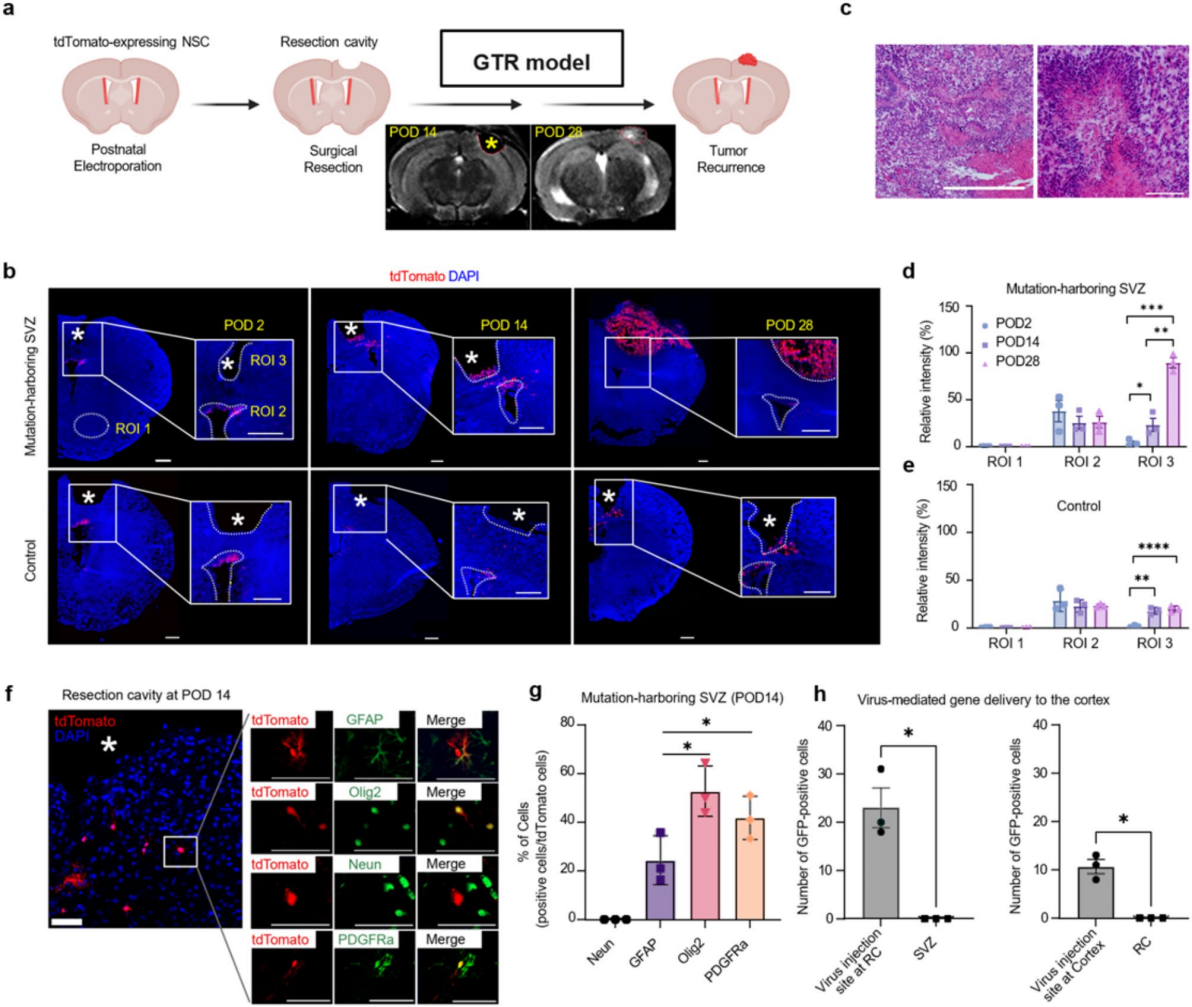
Glioma reconstruction at RC in genome-edited mice carrying cancer mutations in NSCs from the SVZ. **a**, An experimental model used in an attempt to replicate the local reconstruction of glioma at the RC. Initially, a plasmid containing single guide RNA (sgRNA) targeting towards the p53 and Pten genes, along with the expression of Cas9 and Cre recombinase, was injected into the SVZ of a LSL-EGFRviii f/+; LSL-tdTomato f/+ mouse, resulting in the creation of an SVZ-mutated model. A control group was also established, wherein a Cas9/Cre-expressing plasmid containing sgRNA for the lacZ gene was injected into the SVZ of a LSL-tdTomato f/+ mouse. Subsequently, a surgical resection was carried out 28 days post-injection, with the aim of excising distant cortical tissue while minimizing any possible damage to the adjacent white matter. Then, the brains of both the experimental and control groups were harvested, and tissue sections were collected via cryosectioning at various time points following the surgical resection, specifically 2, 14, and 28 days post-resection, referred to as POD2, POD14, and POD28, respectively. (lower panel) Representative MRI images of local recurrent tumor development in RC at POD14 and POD28. Created with Biorender.com. **b**, Representative coronal sections of mice stained with DAPI at 2, 14, and 28 days post-resection in mice of each group (*n* = 3). The asterisk indicates the RC. Images from SVZ-mutated mice show that tdTomato-positive cells were initially localized in the rostral SVZ, where mutations were introduced. Over time, these cells migrate to the RC and proliferate to form a tumor; however, there is no tumor formation in the control group. The white dashed line indicates the SVZ and RC sites. Scale bars, 500 μm. **c**, Representative images of mouse tumor sections stained with H&E. Scale bars, 200 μm. **d-e**, Quantification of tdTomato immunofluorescence intensity (relative to DAPI) in ROI1-3 (Region of Interest) regions at different timepoints post resection of the SVZ-mutated group, compared with the control group. ROI 1 was defined as any random area of the brain excluding the SVZ or RC, ROI 2 as the SVZ region, and ROI 3 as the RC site. *P = 0.0423, **P = 0.0017, **** *P*<0.0001 (*n* = 3 for control and mutant mice at each time point). Error bars represent mean ± s.e.m. **f**, Representative immunostaining images of RC at POD14, stained with GFAP, Olig2, PDGFRa and Neun. Scale bars, 20 μm. **g**, Quantification of lineage marker-positive tdTomato-positive cells at RT at POD14 in mutation-harboring SVZ group. *P = 0.0012(GFAP vs. Olig2). *P = 0.0427 (GFAP vs. PDGFRa). Student’s two-tailed t-test. Error bars represent mean ± s.e.m. **h**, Comparison of GFP positive cell number in RC with virus injection, SVZ, and cortex with virus injection, respectively. *P = 0.0295 (left panel), *P = 0.0181(right panel) (*n* = 3). Student’s two-tailed t-test. Error bars represent mean ± s.e.m

To eliminate the potential influence of residual tumor cells in generating recurrent tumors from our analysis, we optimized our mouse model by performing a surgical resection of the cortex, where we simulated the post-resection status following a gross total resection (GTR) of the primary tumor (GTR model) (Fig. 2a). Briefly, we performed postnatal electroporation of a vector expressing Cas9 and Cre recombinase, along with sgRNAs targeting *Trp53* and *Pten* into the SVZ of LSL-EGFRviii; LSL-tdTomato mice (Supplementary Fig. 4d-f). At four weeks of age, we performed a surgical resection of the distant cortex, while ensuring minimal disturbance to the adjacent white matter and observed the behavior of mutation-harboring SVZ cells migrating to the RC (Fig. 2b). Aligning with these expected findings, 64.7% of the mice (11 out of 17) developed brain tumors surrounding the RC four weeks after surgery. Histological examination of these tumors revealed the classical features of highly proliferative, high-grade glioma (Fig. 2c); by contrast, control mice with SVZ NSCs targeted by sgLacZ exhibited no tumor formation.

To monitor the proliferation and migration of tdTomato-expressing SVZ cells, we selected three regions of interest (ROI), i.e., the SVZ, RC, and distant ventral region to analyse the fluorescent intensity detected in each ROI. Increased proliferation of SVZ cells was detected two days after cortical resection, with these cells appearing within the RC starting at 14 days in both SVZ cells with and without driver mutations (Fig. 2d, e). However, only the mutation-harboring SVZ cells were able to reconstruct tumors within the RC by 28 days (Fig. 2b). To rule out the influence of spatial proximity between the surgical site and the SVZ, we performed surgical resection in the distant caudal brain of the parietotemporal lobe, after which we still observed tumor occurrence at the distant RC (Supplementary Fig. 5). We also conducted immunostaining analyses to characterize the cellular subtypes of the migrating cell population derived from the SVZ (Fig. 2f, g). Notably, Olig2 + oligodendrocyte progenitor cells (OPCs) emerged as the predominant cellular subtype among tdTomato-positive cells migrating to the RC at 14 days post-surgery, suggesting that aberrant proliferation and differentiation into the OPC lineage of mutation-harboring NSCs are essential for recurrent tumor formation (Fig. 2h). Overall, our findings provide valuable insights into the spatiotemporal dynamics underlying GBM recurrence after surgical resection.

### Upregulation of CXCR4 expression in migrating SVZ cells

**Fig. 3:**
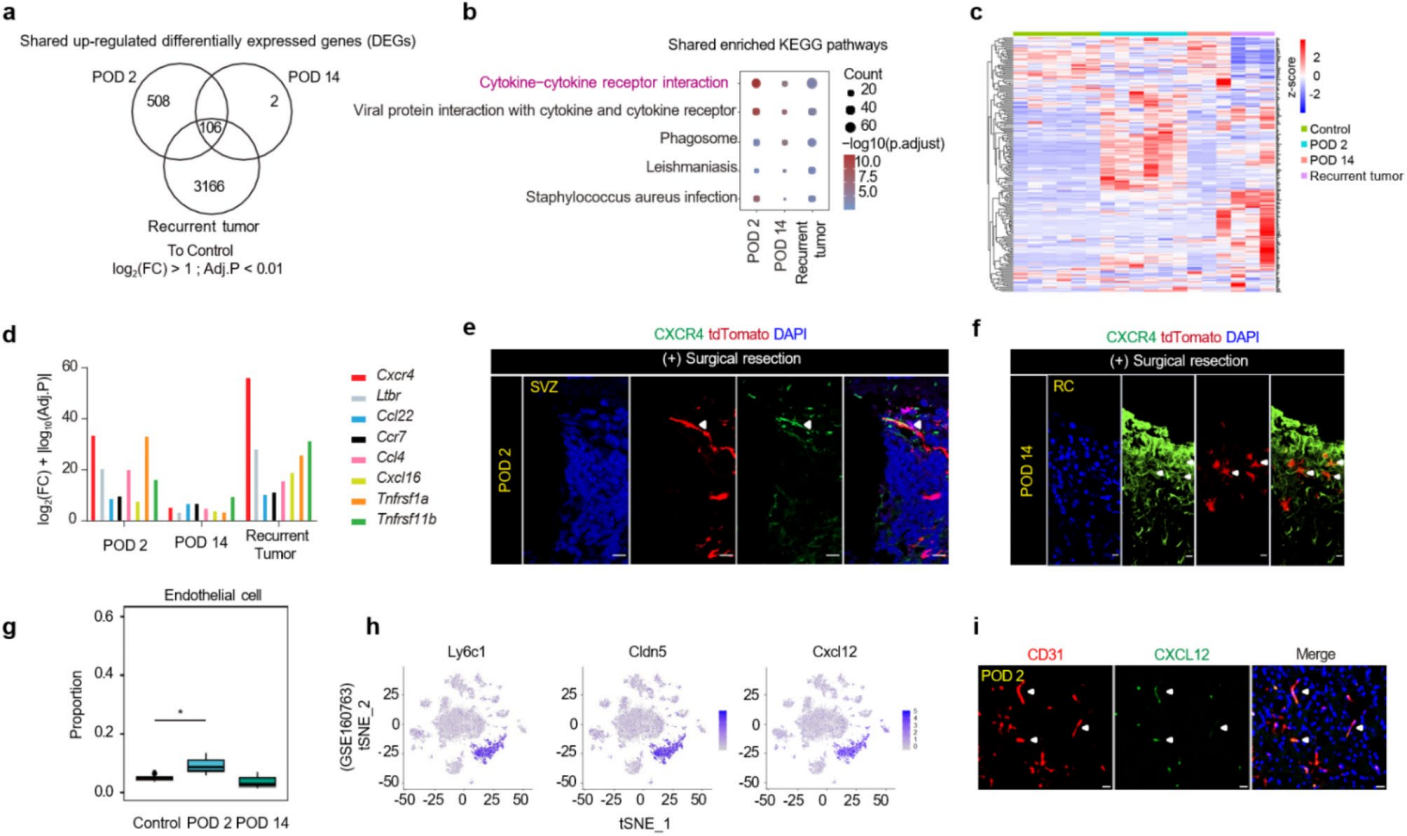
*Cxcr4* was highly expressed at the RC after surgical resection and correlated with migration of SVZ-mutated NSCs. **a**, Venn diagram demonstrating the overlap between upregulated differentially expressed genes (DEGs) detected via the RNA-sequencing analysis of samples from the POD2, POD14, and tumor groups versus the control group samples. **b**, Bubble plot showing the overlap between enriched KEGG pathways detected via the RNA-sequencing analysis of samples from the POD2, POD14, and tumor groups versus the control group samples. **c**, Heatmap showing the expression of the geneset of the cytokine-cytokine receptor interaction pathway in the control, POD2, POD14, and tumor groups. **d**, Comparison of the expression levels of eight genes filtered out through panel d among POD2, POD14, and tumor groups versus control group samples. **e**, Representative immunostaining images of CXCR4 in the mouse SVZ at POD2 after surgical resection. The white arrowheads represent CXCR4-positive cells colocalizing with tdTomato + cells in the SVZ-mutated group. Scale bars, 50 μm. **f**, Representative immunostaining images of CXCR4 in the RC on POD14. The white arrowheads represent CXCR4-positive cells colocalizing with tdTomato + cells in the SVZ-mutated group. Scale bars, 50 μm. **g**, Boxplot showing the proportion of endothelial cells in control, POD2, and POD14 groups after deconvolution of RNA-seq from a mouse model. **h**, t-SNE plots from the public dataset (GSE160763) showing upregulated expression of representative marker genes of endothelial cells (*Ly6c1* and *Cldn5*) as well as Cxcl12 in mice in the traumatic brain injury group. **i**, Representative immunostaining images of CD31 and CXCL12 in the RC on POD2 after surgical resection. Scale bars, 20 μm, and the white arrowheads represent CD31-positive endothelial cells colocalizing with CXCL12. Scale bars, 20 μm

Next, to elucidate the factors involved in the migration and tumor formation of mutation-harboring NSCs in the SVZ, we analysed the transcriptomic gene signatures of the RC. To this end, we collected the tissues surrounding the RC on post-operative days (POD) 2, 14, and 28, and assessed them using RNA-seq analyses. We focused on genes that are consistently upregulated in the RC compared to the normal cortex, and we identified 106 co-upregulated genes across all time points (Fig. 3a). Subsequent KEGG analysis revealed that these genes were enriched in the cytokine cytokine-receptor interaction pathway (Fig. 3b, c). Notably, we identified eight cytokine genes that were significantly co-upregulated in our model: *Cxcr4*,* Ltbr*,* Ccl22*,* Ccr7*,* Ccl4*,* Cxcl16*,* Tnfrsf1a*, and *Tnfrsf11b* (Fig. 3d). Among them, CXCR4 expression was found to be highly elevated in migrating tdTomato-positive cells at the RC, and this was validated through staining of the RC and normal cortex (Fig. 3e, f, Supplementary Fig. 6).

To identify the potential source of CXCL12, we performed deconvolution analysis of our RNA-seq data along with single-cell RNA-seq analysis. Studies have shown that CXCL12 is released by multiple cell types in the brain, including neuronal, glial, and endothelial cells. Deconvolution analysis indicated that the proportion of endothelial cells around the RC was significantly increased at POD2 (Fig. 3g). Single-cell RNA-seq analysis of a brain trauma model (GSE160763) revealed that *Cxcl12* expression was specific to *Cldn5*-expressing endothelial cells (Fig. 3h), and this was further confirmed by the findings of immunostaining analysis showing augmented secretion of CXCL12 in endothelial cells (Fig. 3i). This increased release of CXCL12 surrounding the RC in turn likely facilitates the migration of CXCR4-positive NSCs to this site, thus contributing to tumor recurrence.

### Clinical implications of the CXCR4 pathway in tumor recurrence

**Fig. 4:**
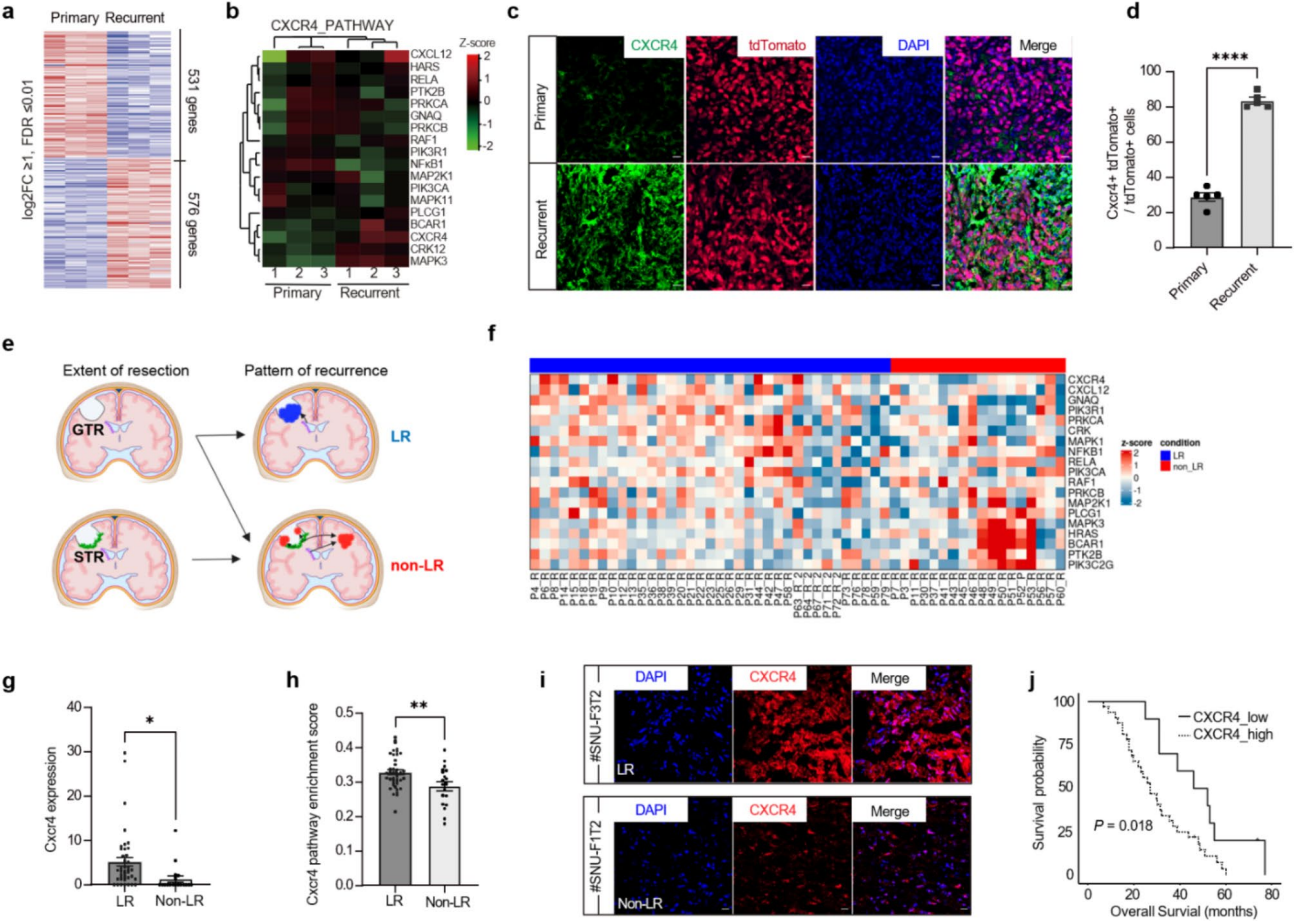
CXCR4 expression in the GBM reconstructed after surgical resection in mice and human GBM patients was increased when compared to primary tumors. **a**, Heatmap of the top 50 differentially expressed genes between primary and recurrent GBM groups. **b**, Heatmap depicting the expression levels of the 18 signature genes between recurrent and primary tumors. **c**, Immunostaining images of *Cxcr4* expression in primary and recurrent GBM tissues of mice, with the lowest panel showing the tumor tissues from the recurrent GBM after primary tumor removal. **d**, Quantification of *Cxcr4* expression levels in each group. Statistical analysis was performed using a two-sided Student’s t-test (****P < 0.0001). Data are presented as mean ± SEM. **e**, Schematic illustration of tumor recurrence patterns following gross total resection (GTR) and subtotal resection (STR). Cases of local recurrence possibly originating from neural stem cells were classified as the LR group, while other cases were categorized as the non-LR group. Created with Biorender.com. **f**, Heatmap showing the expression of the CXCR4 pathway geneset in the LR and non-LR groups. **g-h**, Comparison of CXCR4 expression levels **(g)** and CXCR4 pathway enrichment scores (h) in the LR and non-LR tumor samples. Statistical analysis was performed using a two-sided Student’s t-test (*P < 0.05). Data are presented as mean ± SEM. **i**, Representative immunostaining images of CXCR4 expression in recurrent GBM tissues from human patients (#SNU-F3T2, LR group; #SNU-F1T2, non-LR group). Scale bars, 50 μm. **j**, Overall survival curve of recurrent GBM patients in the LR group stratified by CXCR4 expression

To further investigate the involvement of CXCR4 in recurrent tumor development, we conducted a comparative transcriptomic analysis between primary and recurrent tumors in our mouse model of GBM. Differentially expressed gene analysis identified 576 up-regulated and 531 down-regulated genes in recurrent tumors (Fig. 4a). Based on the results of our time-course transcriptome analysis, we hypothesized that the CXCR4 pathway might be enhanced in recurrent malignancies. Indeed, the expression of genes in the CXCR4 pathways was found to be elevated in recurrent tumors (Fig. 4b). Immunostaining analysis also demonstrated a significant increase in CXCR4 levels in recurrent tumors (*P* < 0.0001) (Fig. 4c, d). Moreover, our GBM recurrence model revealed increased expression of *Cxcr4* and enrichment of CXCR4 pathways in GFP-positive and mixed recurrent tumors, suggesting that CXCR4 plays a critical role as a key regulator in SVZ-derived tumor recurrence (Supplementary 7).

To further explore the potential link between CXCR4 signaling and the SVZ-driven recurrence phenotype in GBM, we closely examined the pattern of tumor recurrence and conducted RNA-seq analysis on 55 recurrent tissue specimens. Given that CXCR4 is a key regulator of the migration and repopulation of mutation-harboring SVZ cells in the RC, we hypothesized that CXCR4 expression might be particularly elevated in cases of local recurrence (LR) within the RC following total removal of primary tumors via GTR (Fig. 4e). Patients who experienced tumor recurrence at the RC after complete resection of primary tumor were designated as the LR group, with this subgroup used to investigate tumors potentially driven by SVZ NSCs. Conversely, other types of recurrence, including those from the residual primary tumor after subtotal resection, were classified into the non-LR group. Comprehensive transcriptome analysis revealed that CXCR4 pathway-related genes were highly expressed in the LR group (Fig. 4f-h). *CXCR4* expression was significantly elevated in the LR group, both in RNA-seq data and immunostaining of recurrent tumor tissues (Fig. 4i). Notably, we observed a significant correlation between high CXCR4 expression levels and poorer overall survival (OS) outcomes in LR patients (*P* = 0.018) (Fig. 4j), thus indicating the prognostic relevance of CXCR4 in distinguishing distinct recurrence patterns and their associated clinical trajectories. This finding underscores the potential utility of CXCR4 as a prognostic biomarker for stratifying GBM patients based on their recurrence risk as well as for guiding personalized treatment strategies.

### Therapeutic potential of targeting the CXCR4/CXCL12 pathway

**Fig. 5:**
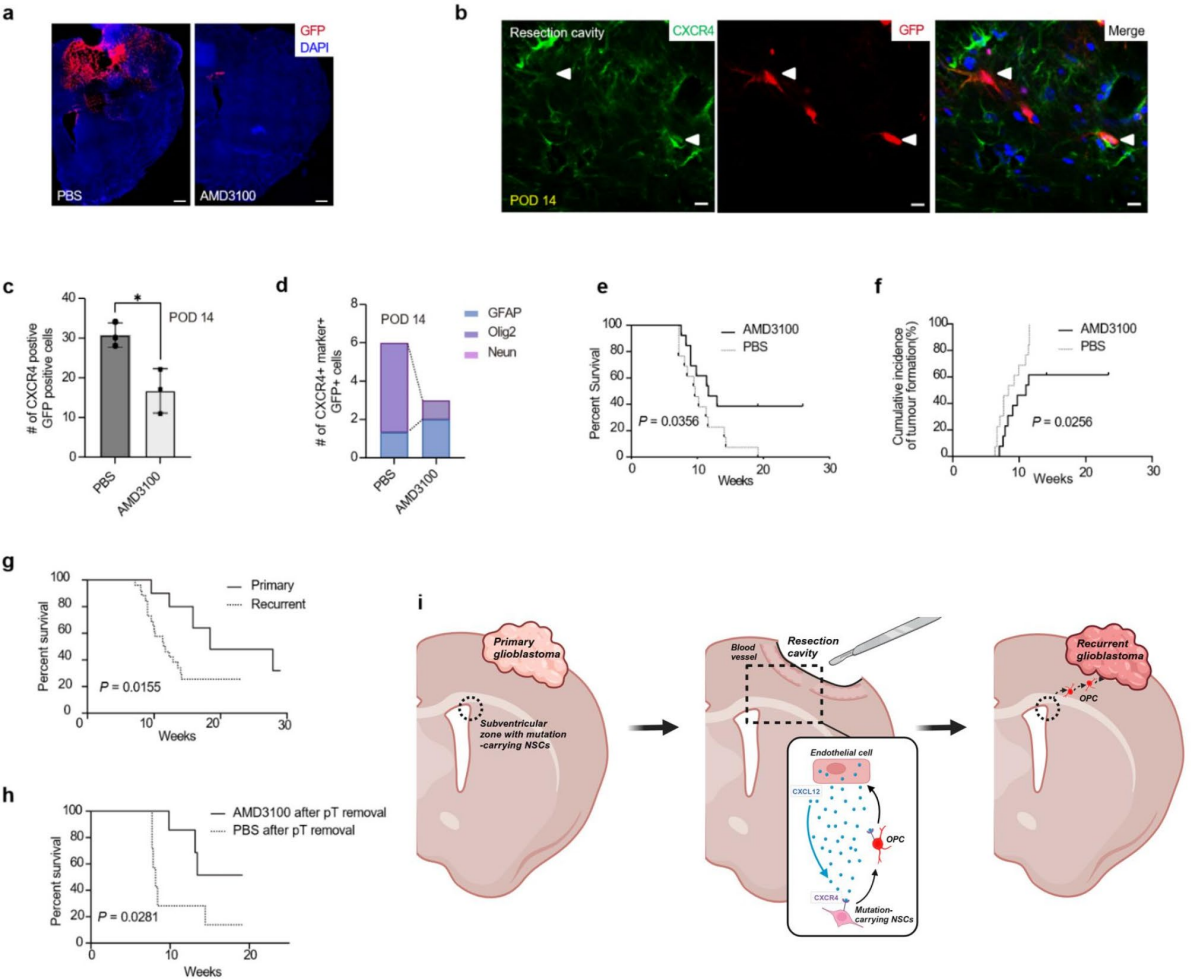
Treatment via CXCL12/CXCR4 blockade decreased the number of immigrating OPC lineage cells and improved survival in mice. **a**, Representative images of local reconstructed tumor development treated with AMD3100, a CXCR4 inhibitor, while the control group was treated with PBS on POD28 following surgical resection. Scale bars, 500 μm. **b**, Representative images of GFP + cells around RC after their migration from the SVZ at POD14 of surgical resection. The white arrowheads represent CXCR4 colocalizing with GFP + cells. Scale bars, 50 μm. **c**, Quantification of the number of CXCR4 + cells in immigrating GFP + cells around the RC at POD14 of surgical resection in AMD3100 treatment versus PBS group (*n* = 3 per group). Statistical analysis was performed using a two-sided Student’s t-test (*P < 0.05). Data are presented as mean ± SEM. **d**, Quantification of the number of CXCR4 + cells that showed double-staining with GFAP, OLIG2, and NeuN in AMD3100 treatment, versus PBS group at POD14 after surgical resection. **e**, Kaplan-Meier survival analysis of mice (*n* = 13 per group) in AMD3100 treatment, versus PBS group; *P* = 0.0356 (log-rank test). **f**, Cumulative incidence of tumor in mice (*n* = 13 per group) in AMD3100 treatment, versus PBS group; *P* = 0.0256 (log-rank test). **g**, Kaplan-Meier survival analysis of mice (*n* = 11 per group) without surgical resection (primary tumor), and of mice after surgical resection (recurrent tumor); *P* = 0.0155 (log-rank test). **h**, Kaplan-Meier survival analysis of mice (*n* = 7 per group) in AMD3100 treatment group after transplanted primary tumor removal versus PBS group; *P* = 0.0281 (log-rank test). **i**, Graphic illustration showing the mechanism by which CXCL12/CXCR4 axis-mediated tumor initiate NSCs in SVZ immigrating to RC in glioblastoma recurrence. Created with Biorender.com

Following complete resection of GBM, the CXCR4/CXCL12 pathway has emerged as a promising therapeutic target to improve patient outcomes, particularly in preventing SVZ-driven recurrence [29]. Although radiotherapy and temozolomide have shown efficacy in treating GBM, the high rate of local recurrence suggests a need for additional novel therapeutic approaches. To investigate the effects of pharmacological intervention targeting the CXCR4/CXCL12 chemokine axis, we treated our modified mouse model for GBM recurrence with the CXCR4 blocker Plerixafor (AMD3100). Following cortical resection, AMD3100 was administered twice at a dose of 1.25 mg/kg via intraperitoneal injection. Remarkably, the treated group did not exhibit tumor formation within the RC at POD28 (Fig. 5a). Although AMD3100 treatment did not completely inhibit the migration of SVZ cells, it significantly reduced the number of mutation-harboring SVZ cells expressing CXCR4 and decreased the differentiation of these cells into Olig2 + OPCs (Fig. 5b-d). Notably, blockade of the CXCR4/CXCL12 axis led to reduced incidence of tumor recurrence and prolonged survival rates in both GBM recurrence and GTR models (Fig. 5e-h).

To further elucidate the impact of CXCR4 blockade on NSC differentiation, we generated neurospheres from the SVZ of LSL-EGFRviii f/+; LSL-Cas9-GFP f/+ mice in response to the introduction of cancer-driving mutations (Supplementary Fig. 8a). Cells were treated with AMD3100 for 7 days, followed by immunostaining and an analysis of lineage differentiation (Supplementary Fig. 8b). Interestingly, AMD3100 treatment inhibited OPC differentiation, particularly favoring differentiation toward astrocytes that are positive for glial fibrillary acidic protein (GFAP) (Supplementary Fig. 8c, d). This observation suggests that CXCR4 blockade may influence the differentiation capacity of mutation-harboring NSCs, which is a critical step in tumor reconstruction alongside migration (Fig. 5i). Altogether, our findings provide valuable insights into the molecular mechanisms underlying tumor progression while also highlighting the therapeutic potential of targeting the CXCR4/CXCL12 axis in preventing local recurrence of GBM.

## Discussion

GBM is a primary brain tumor that continues to have a poor prognosis despite the range of treatment options available, including surgical resection, radiation therapy, and chemotherapy [1, 4, 30]. Previous research has established that cancer-driving mutations in SVZ NSCs can be the cellular origin of GBM [12, 14, 15]. Our findings provide novel evidence suggesting that GBM recurrence may result from the repetitive and cyclical evolution of the remaining tumor-initiating NSCs located in the distant SVZ after primary tumor resection. The persistence of mutation-harboring NSCs represents a potential therapeutic target for preventing recurrence of this challenging disease.

Several longitudinal studies have utilized paired primary and recurrent tumor specimens from GBM patients. In terms of recurrence, approximately 40–60% of recurrences showed divergence of somatic mutations from primary tumors [26–28, 31]. The linear evolution model fails to explain this genetic divergence, supporting a branched evolutionary trajectory in which early progenitor clones—present even before diagnosis—drive recurrence independently of the dominant primary clones. We hypothesized that this branched evolution trajectory might be associated with the involvement of distant, mutation-harboring SVZ cells in tumor recurrence [26–28, 31]. To explore this possibility, we analyzed 10 patients with four matched samples: blood, tumor-free SVZ, primary, and recurrent tumors. In 60% of cases, recurrent tumors shared a somatic mutation with both tumor-free SVZ and primary tumors, while primary-private mutations were not retained in the recurrent tumors. Instead, the recurrent tumor acquired additional oncogenic mutations independent of the primary tumors. These findings suggest that the mutation-harboring SVZ may serve as a potential cellular reservoir of the early progenitors that evolve into recurrent tumors, distinct from the primary dominant clones, alongside residual tumor cells. However, the inherent heterogeneity of GBM and the possibility of sampling bias must be carefully considered especially whether other early progenitors may drive the recurrence. The spatial and genetic diversity of GBM could result in either the underrepresentation or overrepresentation of certain subclonal populations within tumor samples, contributing to variability in observed clonal evolution patterns. To address these challenges, future studies should incorporate multi-sectional sampling strategies to better account for tumor heterogeneity and further clarify the genetic linkage between the SVZ and recurrent tumors.

To elucidate the underlying mechanisms of the tumor-initiating NSCs in the SVZ responding to surgical resection, we collected time-course samples from the RC area in a genome-editing mouse model and performed RNA sequencing. Notably, cytokine levels began increasing as early as POD2 and remained elevated from them, which is consistent with previous reports indicating that chemokines play a crucial role in tumor progression and metastasis [32, 33]. Among these genes, CXCR4 was found to be the most upregulated chemokine-associated gene during recurrence CXCR4 is known to drive the migration of NSCs toward injury sites after traumatic brain injury [19, 34]. Our study suggests that increased CXCR4 expression in mutation-harboring NSCs from the SVZ contributes to GBM recurrence. In addition to previous reports on CXCR4 expression associated with GBM [35–37], our results highlight that CXCR4-positive SVZ NSCs should be examined as a potential target for therapeutic intervention, particularly in the context of local recurrence after surgical resection.

Our findings also indicate that CXCR4 is involved in the differentiation of mutated NSCs toward the OPC lineage. This is consistent with previous observations showing that CXCR4 promotes the differentiation of OPCs into oligodendrocytes during remyelination in injured adult CNS [38]. The OPC lineage was found to be the dominant cell type involved in glioma reconstruction at the RC in our mouse model, suggesting that mutated NSCs migrate specifically to the RC through OPC lineage. Previous studies have reported that mutated NSCs in the SVZ contribute to glioma through aberrant growth of the OPC lineage [32, 34, 39]. Thus, high CXCR4 expression is associated with NSC differentiation into OPCs and migration to the RC, ultimately leading to glioma reconstruction at this site. Moreover, CXCL12/CXCR4 blockade by AMD3100 reduced OPC lineage cell markers in vitro, supporting the idea that increased CXCR4 expression promotes NSC migration and differentiation. CXCL12/CXCR4 inhibition also reduced tumor development and mortality rates in mice with mutated residual NSCs in the SVZ. Notably, high CXCR4 levels were found in local recurrent tumors from GBM patients after GTR, underscoring that CXCL12/CXCR4 is a promising target for GBM treatment.

Strong and specific CXCR4 expression was observed in recurrent tumor cells (Supplementary Fig. 9), suggesting its critical role in local recurrence. Nevertheless, immune cell-derived CXCR4 expression may contribute to the high expression of CXCR4 observed in the bulk RNA sequencing of the locally recurrent tumor samples [25, 43, 44]. To elucidate the complex interplay between tumor cell-derived and immune cell-derived CXCR4 in local recurrence originating from SVZ NSCs, further investigations are warranted. Another important consideration is the interaction of CXCL12 with its alternative receptor, ACKR3, which is known to be signaling-defective and functions primarily as a scavenger receptor [45, 46]. In addition to the role of CXCL12/CXCR4 signaling in tumor recurrence, ACKR3 may modulate CXCL12 availability or signaling dynamics within the tumor microenvironment, influencing recurrence pathways indirectly. Further investigation into this scavenger receptor and its potential impact on CXCL12-mediated mechanisms may provide additional insights into the complexity of tumor relapse processes.

Our patient sequencing data could also support an alternative scenario, where an early GBM clone migrates to the SVZ, while low-level somatic mutations associated with various brain disorders have been consistently observed [40–42]. Although histological examination and deep sequencing were employed to detect low-frequency shared and private clones, these methods may not completely rule out the possibility of micro-invasion by early GBM clones, as indicated by our human genetic data analysis. Previous studies have reported SVZ can serve as a hidden reservoir, allowing primary tumor cells to migrate to the SVZ during tumor progression, particularly in patient-derived xenograft (PDX) models [43–45]. In addition to the mutation-carrying NSCs implicated in recurrence in this study, the subclone of primary tumor initially invading SVZ might contribute to local recurrence through bidirectional migration between SVZ and primary sites. While this hypothesis also remains plausible, it ultimately underscores the need for further studies to elucidate the role of the SVZ as a distant source and to explore strategies for suppressing recurrence.

## Conclusion

In summary, our study provides evidence showing that SVZ NSCs may serve as a viable target for preventing GBM recurrence following surgical resection, which could facilitate the development of novel treatment strategies for glioma patients. We emphasize the importance of understanding the residual origins of solid cancers after primary treatments and their interrelation with these treatments. Further research is needed to develop new therapies that effectively target these residual origins of cancer.

## Materials and methods

### Human biospecimens

This study was approved by the Institutional Review Board of Seoul National University Hospital (IRB#H-1907-103-1048 and #H-0507-509-153) and Yonsei University Severance Hospital (IRB# 4-2021-1319). Recurrence samples were prospectively collected between 2013 and 2022 with at least three matched specimens obtained from each patient, including: (i) pathologically normal SVZ tissue, (ii) primary tumor tissue after gross total resection, and (iii) blood. Sample collection was conducted following our previously described protocol [12]. H&E-stained slides were carefully reviewed by a neuropathologist to assess the presence or absence of tumor infiltration into the SVZ. Patients with evidence of suspicious SVZ infiltration were excluded following mutational analysis (Supplementary Fig. 1h, i). From this initial cohort, 10 patients who experienced tumor recurrence were selected for further analysis through deep sequencing of their recurrent tumor samples. Each selected patient exhibited features of clonal evolution at the time of their primary tumors, as described in the Results section. Recurrence samples were not collected from patients who either did not experience recurrence during follow-up or were ineligible for re-surgery due to the salvage nature of their treatment.

For RNA sequencing, we collected recurrent GBM tissues from 55 patients with confirmed cases of tumor recurrence. Among these, 44 samples were obtained from Seoul National University Hospital, and 11 samples were collected from Yonsei University Severance Hospital. All samples were collected with the informed consent from the participants. To ensure the accuracy and validity of our findings, the extent of tumor resection was assessed using pre-operative and post-operative magnetic resonance imaging (MRI) scans. Survival times were calculated from the date of the initial surgery to evaluate the effectiveness of the surgical interventions and their impact on patient outcome.

### Longitudinal analysis of four matched samples

Genomic DNA was extracted from fresh frozen samples using the QIAamp DNA Mini Kit (Qiagen, #51304). For peripheral blood samples, DNA extraction was performed using the QIAamp DNA Blood Mini Kit (Qiagen, #51104). DNA yield was quantified using the Qubit dsDNA Quantification Assay Kit (Invitrogen, #Q332391) on a Quant 4 Fluorometer (Invitrogen). A custom NGS panel was designed to include 400 amplicons ranging from 125 to 175 bp, which were distributed across two primer pools. This panel covered 100.08 kb and targeted a 200 bp region of the *TERT* promoter, as along with coding and untranslated regions of 14 cancer driver genes that are frequently mutated in GBM [46, 47]. The targeted gene hybrid sequencing was designed and manufactured by Celemics Inc. (Seoul, Korea). The libraries were prepared according to the manufacturers’ protocol, and the final libraries were sequenced on a MiSeq Dx sequencer (Illumina, CA) by Sovargen (Daejeon, Korea).

BAM files were generated from FASTQ files following the GATK Best Practices workflow established by the Broad Institute. Sequenced reads were mapped to the human reference genome (hg19) using the BWA-MEM algorithm. Duplicated reads were removed using Picard, and base quality score recalibration was applied to correct for systematic errors in the base quality score. Somatic base substitutions and short indels were identified using Mutect2, with matched blood samples used as controls; only variants that passed the “PASS” filter were included in our final analysis. All single nucleotide variations (SNVs) were annotated using the Ensemble Variant Effect toolset. To ensure accuracy and exclude false positive events, all mutations were visually inspected using the Integrative Genomics Viewer (IGV). Moreover, LICHeE (https://github.com/viq854/lichee) was used to reconstruct longitudinal-sample cell lineage trees and infer the sub-clonal composition of the samples based on the variant allele frequencies of somatic single nucleotide variants.

### Whole genome sequencing and targeted amplicon sequencing

DNA libraries for whole-genome sequencing (WGS) were prepared from 1 µg of genomic DNA using the Truseq DNA PCR-Free Library Prep Kit (Illumina). Sequencing was carried out on the Illumina NovaSeq 6000 platform, and average coverages of 60× for tumors and 30× for matched blood tissues were ultimately achieved. Reads were aligned to the human reference genome (GRCh37) using the BWA-MEM algorithm. Duplicated reads were filtered out using Picard, while base substitution and short indels were identified with MuTect2 and Strelka2. Additionally, segment copy-number profiles for the whole-genome sequenced samples were estimated using the Sequenza [48] algorithms, with matched blood samples used as controls. To ensure accuracy and exclude false positive events, all mutations were visually inspected using the Integrative Genomics Viewer (IGV).

For validation, deep targeted amplicon sequencing was performed using four matched specimens for each patient. Primers were designed using the Primer3 software (https://primer3.ut.ee/), and the primer sequences are listed in Supplementary Table 1. The target region was amplified by PCR and high fidelity PrimeSTAR GXL DNA polymerase (Takara, Shiga, Japan). The MiSeq Reagent Micro Kit v2 (300 cycles) were used for library preparation and sequenced on a MiSeq Dx sequencer (Illumina, CA) by Sovargen (Daejeon, Korea).

### Cre-expressing constructs to model p53, Pten mutations

AAV: ITR-U6-sgRNA(backbone)-pCBh-Cre-WPRE-hGHpA-ITR was provided as a gift from Feng Zhang (Addgene plasmid #60229; http://n2t.net/addgene:60229; RRID: Addgene_60229). pAAV-U6sgp53-U6sgbbSapI-GFAPCre was provided as a gift from Sidi Chen (Addgene plasmid #100275; http://n2t.net/addgene:100275; RRID: Addgene_100275). gRNAs targeting p53 (sgP53) and Pten (sgPTEN) were designed as previously described^[12]^. The sequences for the targeting sgRNA are as follows: sgP53 5’-TAATAGCTCCTGCATGG-3’, sgPTEN 5’-GGTCAAGATCTTCACAGA-3’. Oligonucleotides containing these sgRNA sequences were synthesized by Cosmogenetech and annealed in vitro using a thermocycler.

To create a single vector containing sgRNAs targeting p53, Pten, and Cre recombinase under the control of the GFAP promoter, we first amplified the GFAP promoter from pAAV-U6sgp53-U6sgbbSapI-GFAPCre plasmid, after which replaced the pCBh promoter in the pU6-sgRNA(backbone)-pCBh-Cre-WPRE-hGHpA plasmid with the GFAP promoter. Next, pU6-sgP53 and pU6-sgPTEN were amplified, and the pU6-sgRNA(backbone)_pGFAP-Cre-WPRE-hGHpA was modified to include both sgP53 and sgPTEN, ultimately resulting in the pU6-sgP53-pU6-sgPTEN_pGFAP-Cre-WPRE-hGHpA plasmid (referred to as sgTP-GFAP-cre).We also generated the sgLacz-pGFAP-Cre plasmid by inserting the U6-sgRNA(backbone)_pGFAP-Cre-WPRE-hGHpA sequence.

### Mouse care and information

All experiments were approved by the Institutional Animal Care and Use Committee in Seoul National University Hospital (SNUH-IACUC). The animals were housed in a facility accredited by AAALAC International (#001169) and were maintained in accordance with the Guide for the Care and Use of Laboratory Animals 8th edition, NRC (2010). The LSL-tdTomato (007914), LSL-Cas9 (25263330), and LSL-EGFRviii (19196966) mice [49] were purchased from the Jackson Laboratory and maintained on a C57BL/6 strain and FVB strain background. The mice were housed in isolator cages where they had free access to food and water in a quiet, specific-pathogen-free room that was maintained at a controlled temperature of 23 °C on a 12-hour light-dark cycle. The veterinarians and the researchers carried out routine health assessments. Disease-specific survival endpoints were defined as either the natural death of the mice or the meeting of euthanasia criteria as specified under the IACUC protocol.

### In vivo electroporation

Postnatal day 0 (P0) to P2 mice pups were anesthetized on ice for over 5 min. The injection site was defined as the midpoint of an imaginary line connecting the lambda and the upper-left corner of the eye. A mouth-controlled microinjection capillary was assembled for use, and the capillary was slowly lowered 4 mm down into the right lateral ventricle. Plasmid solution supplemented with 1% Fast Green dye was injected, and the capillary was retracted 5 s after the completion of the injection. Following successful injection, 5-mm tweezer electrodes (45–0489, BTX-Harvard apparatus) were positioned on each side of the head, and five electrical pulses (100 V, 50 ms duration, 950 ms intervals) were delivered using an ECM830 electroporator (BTX-Harvard apparatus). After electroporation, the pups were placed on a 37 °C heating pad until they began to move, at which point they were returned to their mother.

### Surgical resection

To perform surgical resection in four-week-old mice, anesthesia was induced through intraperitoneal injection of a ketamine-xylazine mixture. The mice were then securely positioned in a stereotaxic frame (Stoelting) for precise localization of the surgical site. A longitudinal skin incision was made, and a hand-held drill was used to create a 2 mm diameter craniotomy centered at 2 mm posterior to the bregma and 2 mm lateral to the midline. Cortical injury was induced using a flat pipette tip attached to a vacuum pump (VACUSIP, INTEGRA Biosciences), while care was taken to preserve the integrity of the corpus callosum. Following the resection, the mice were placed on a heating plate set to 37 °C until they fully recovered from anesthesia. The animals were then returned to their cages for post-operative care and monitoring.

### Stereotaxic injection of AAV5 into cortex after surgical resection

To construct adeno-associated virus (AAV) targeting astrocytes [50], we co-transfected HEK293T cells with the pAAV5 capsid plasmid, a virus assembly helper plasmid (pAd deltaF6, obtained from the UPENN Vector Core), and target plasmids using polyethylenimine (1 mg/ml). Before transfection, the fetal bovine serum (FBS; Gibco)-containing DMEM (Gibco) was replaced with serum-free media. Six hours after transfection, the media was replaced again with FBS-containing DMEM, and the transfected cells were incubated for 72 h. The cells were then harvested, resuspended in a solution of 50% fresh DMEM and 0.04% DNase I (Worthington) in nuclease-free water, and lysed through three freeze-thaw cycles. Cellular debris was separated from the AAV-containing supernatant by centrifugation, at which point the supernatant was collected. AAV particles were then purified using polyethylene glycol-mediated precipitation^[39, 50]^.

For stereotaxic injection, four-week-old mice were anaesthetized through intraperitoneal injection of a mixture with ketamine and xylazine, after which they were positioned in a stereotaxic frame (Stoelting). Hamilton syringes were inserted through the craniotomy using the stereotaxic apparatus and positioned unilaterally at the brain’s surface. The injection coordinates were 0.8 mm caudal, -1 mm lateral, and − 1 mm ventral to the bregma. In total, 100 nL of virus solution was injected at a flow rate of 25 nL/min using a syringe pump (KD Scientific Inc.). Following injection, the syringe was retracted slowly after a 3-minute delay. Virus injections were performed at the RC margin either immediately after surgical resection or 2 weeks prior to surgery. After the procedure, mice were placed on a 37 °C heating pad until they fully recovered and then returned to their cages.

### In vivo treatment with AMD3100

CXCL12/CXCR4 chemokine axis was pharmaceutically inhibited using Plerixafor (AMD3100, Sigma, A5602), a specific CXCR4 blocker. Following surgical resection, mice received intraperitoneal injections of AMD3100 (1.25 mg/kg) or PBS twice daily, with a 6-hour interval between injections. After the final injection, the mice were sacrificed, and their brains were harvested for further anaysis.

### Sphere culture and in vitro analysis of differentiation

GBM tumorspheres were generated from tumors induced in LSL-EGFRviii f/+; LSL-tdTomato f/+ mice using the pU6-sgP53-pU6-sgPTEN_CBh-Cas9-P2A-Cre plasmid. These tumorspheres were cultured in Neural Stem Cell media, consisting of DMEM/F12 (Corning, 0-090-CV) supplemented with B27 (50X, Gibco, 17504044), Penicillin-Streptomycin(Gibco, 15140122), and recombinant human EGF (Novoprotein, C046) and bFGF (Novoprotein, C029), each used at a final concentration of 20 ng/ml. Tumorspheres were dissociated with Accutase (Gibco, A1110501), plated at a density of 2 × 10^5^ cells/ml, then passaged every 4–5 days.

SVZ spheres (neurospheres) were generated from mutated SVZ induced in LSL-EGFRviii f/+; LSL-Cas9-GFP f/+ mice using the pU6-sgP53-pU6-sgPTEN_pGFAP-Cre-WPRE-hGHpA plasmid and cultured in Neural Stem Cell media. They were maintained under the same conditions as those described for the GBM tumorspheres.

For neurosphere differentiation analysis, cells were dissociated with Accutase and plated with a density of 40,000 cells per dish, where each dish had been pre-coated with 20 µg/mL Poly-L-ornithine and 10 µg/mL Laminin (Sigma, L2020, and P4957) in Neural Stem Cell media with a reduced concentration of Fetal Bovine Serum (FBS, 1%). AMD3100 (25 µM) or PBS was added to the cells every 72 h. After 7 days of treatment, the cells were immunostained and processed for lineage differentiation analysis.

### Orthotopic implantation of GBM tumorshphere cells and surgical removal of the primary tumor

The tdTomato + tumorsphere cells derived from LSL-EGFRviii f/+; LSL-tdTomato f/+ mice were in the culture for three to four days, and the cells were collected and dissociated into a single cell suspension. The cells were resuspended in a small volume of DMEM/F12 to a concentration of 10^5^ cells/µl, and 1 µl of this suspension was used for stereotaxic injection. The tdTomato + tumor cells were orthotopically implanted into three-week-old LSL-EGFRviii f/+; LSL-Cas9-GFP f/+ mice that had been previously injected with sgTrp53-sgPTEN-GFAP-cre plasmid and electroporated at P0-P2. One week after implantation, bulky primary tumors developed and were surgically resected, as described previously. The mice were monitored daily for signs of tumor burden, and moribund mice were sacrificed. The brains were then processed for histological analysis.

### Image analysis of mouse brain

Mice under each condition were sacrificed and perfused with phosphate-buffered saline followed by 4% paraformaldehyde (PFA). The brains were then harvested, fixed in 4% PFA, and cryoprotected overnight in 30% sucrose. At this point, the tissues were embedded in OCT compound (3801480, Leica Biosystems) and stored as frozen blocks at − 80 °C. Cryostat sections were cut at a thickness of 25 μm and mounted on glass slides. For H&E staining, sections were cut at a thickness of 4 μm. Immunohistochemistry was performed using the following antibodies: Mouse antibody to Nestin (1:200 dilution; MAB5326, Merck Millipore), rabbit antibody to GFAP (1:500 dilution; Z0334, DAKO), rabbit antibody to oligodendrocyte transcription factor 2 (OLIG2; 1:500 dilution; AB9610, Merck Millipore), rat antibody to platelet-derived growth factor receptor α (PDGFRα; 1:200 dilution; 14-4321, eBioscience), rabbit antibody to S100β (1:500 dilution; ab52642, Abcam), rat antibody to myelin basic protein (MBP; 1:500 dilution; MAB386, Merck Millipore), rabbit antibody to Ki67 (1:500 dilution; ab15580, Abcam), rabbit antibody to NeuN (1:500 dilution; ab104225, Abcam), mouse antibody to C-X-C chemokine receptor type 4 (CXCR4; 1:500 dilution; sc-53534, Santa Cruz Biotechnology), and rabbit polyclonal antibody to CXCL12 (1:500 dilution; ab25117, Abcam), Rabbit antibody to CD31 (1:500 dilution; ab222783, Abcam). Nuclear staining was performed using DAPI that had been included in the mounting solution (P36931, ThermoFisher). Images were captured using a Zeiss LSM800 confocal microscope with Z-stacks set at a step size of 1.5 μm. Fluorescence intensities, reflecting the distribution of fluorescent reporter-positive cells were quantified by converting them into grey values using ImageJ software (http://rsbweb.nih.gov/ij/).

### Bulk RNA sequencing

Frozen brain tissue samples from patients with primary and recurrent GBM were dissected, and the tissue samples were rapidly flash-frozen in liquid nitrogen and stored at -80℃ to preserve their integrity. RNA was extracted from the tissue samples using the QIAGEN RNeasy Micro Kit. For sequencing and analysis, cDNA synthesis, library preparation, and sequencing were conducted by technicians at Macrogen. Sequencing was performed using the Illumina Novaseq 6000, and it ultimately yielded 80 million paired-end reads per sample (40 million in each direction). Reads were aligned to human (UCSC hg38) and mouse (mm10) reference genomes using HISAT2 (v.2.2.0) [51], with annotations provided by the Gencode GTF file.

Gene expression quantification was performed using featureCounts from Subread (v1.6.4) [39]. The resulting cell count files were combined into a matrix using a custom Python script (Python 3.6), while raw read counts were filtered to only include genes with at least ten counts across all samples. DESeq2 (v.1.22.2) [52] was used to compute the size factors for each sample and perform variance stabilizing transformation. Batch correction was applied to account for both technical and biological variables. The variance stabilizing-transformed RNA-seq data served as input for clustering analysis. Differential gene expression was assessed using DESeq2, with batch status incorporated as a covariate in this analysis. Upregulated genes were identified based on a Log2Fold Change (FC) greater than 1 and an FDR less than 0.01.

Gene set enrichment analysis (GSEA) (v.3.0) [53] was performed on genes ranked by differential expression, with pathways obtained from the Molecular Signatures Database (MSigDB). Pathways were filtered to include those with a minimum of 15 genes and a maximum of 500 genes. The R package “clusterProfiler” (v3.10.1) [54] was also utilized for GSEA of the Gene Ontology (GO) terms, including biological processes (BP), cellular components (CC), and molecular functions (MF) of differentially expressed genes (DEGs). The five most significant biological processes, with an FDR less than 0.05 and a log2FC less than or equal to 1, were reported. KEGG pathway enrichment analysis of DEGs was also conducted, and the results were visualized in the form of bar plots showing the top 20 pathways.

### Single cell RNA sequencing

Single-cell RNA sequencing data from murine traumatic brain injury were obtained from the GEO database (GSE160763). Clustering and cell type annotation were performed while following previously established protocols [55]. Differential expression analysis for each cell type was conducted using the Wilcoxon rank-sum test, as implemented in the “FindMarkers” function of the Seurat package (v.4.0.4). The expression levels of *Cxcl12* were visualized using tSNE plots to illustrate cellular distribution.

### Deconvolution

Cell fractions from the bulk RNA-seq data for mouse GTR model were enumerated using the CIBERSORTx online platform (https://cibersortx.stanford.edu/). We constructed the reference dataset [55] based on the expression profiles of 2000 highly variable genes in attempt to minimize noise stemming from low-quality genes. Using the single-cell reference file, we followed the instructions for CIBERSORTx while using default parameters to analyze the cellular composition.

### Statistical analyses

Data are presented as a mean ± s.e.m. Results were analyzed with a t-test, Manny-Whitney U test, or Fisher’s exact test where appropriate using GraphPad Prism version 9.4.1 (Graph-Pad Software, Inc.). Tumor accumulated incidence and survival data for mice were analyzed using Kaplan–Meier analysis. All P values less than 0.05 were considered to be statistically significant. All experiments related to the use of animals or the source of cells were subjected to randomization. Sample sizes were predetermined based on the variability found in preliminary and similar experiments. Researchers were not blinded to allocation during experiments or outcome analysis.

## Electronic supplementary material

Below is the link to the electronic supplementary material.

Supplementary Material 1

## Data Availability

No datasets were generated or analysed during the current study.
