# Supplementary material for: Distant origin of glioblastoma recurrence: neural stem cells in the subventricular zone serve as a source of tumor reconstruction after primary resection
Source: Mol Cancer. 2025 Mar 4;24:64. doi: 10.1186/s12943-025-02273-2 (PMC11877783; doi:10.1186/s12943-025-02273-2)
Supplement: Supplementary file 1 — Supplementary Material 1 [file 12943_2025_2273_MOESM1_ESM.docx]

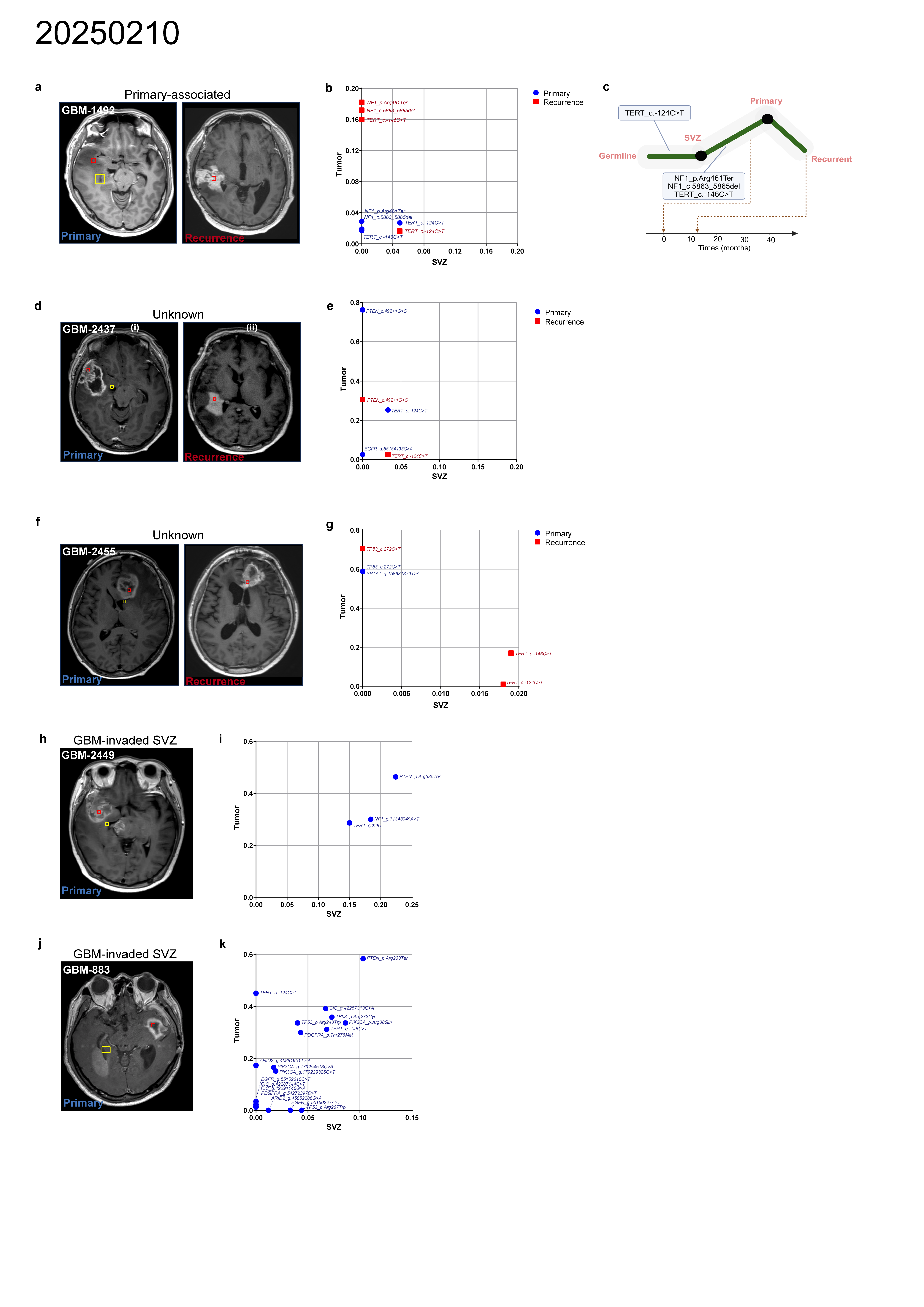


**Supplementary Fig. 1** Mutation profiles and evolutionary patterns in recurrent glioblastomas.

**a-c**, Preoperative and recurrent brain MRI images, variant allele frequency (VAF) scatterplots of mutations, and corresponding tumor phylogenetic evolutionary trees for patient GBM-1492, classified under the primary-associated recurrence group. Sampling sites are marked with boxes: red boxes indicate primary and recurrent tumors, and yellow boxes represent the SVZ. **d-g,** MRI images, and VAF scatterplots of mutations for patients GBM-2437 and GBM-2455, categorized under the unknown recurrence group. **h,** Preoperative MRI image of GBM-2449, with sampling sites indicated by boxes: red boxes denote primary tumor, and yellow boxes represent the subventricular zone (SVZ). **i,** VAF scatterplots of mutations for GBM-2449, indicating suspected GBM invasion into the SVZ. **
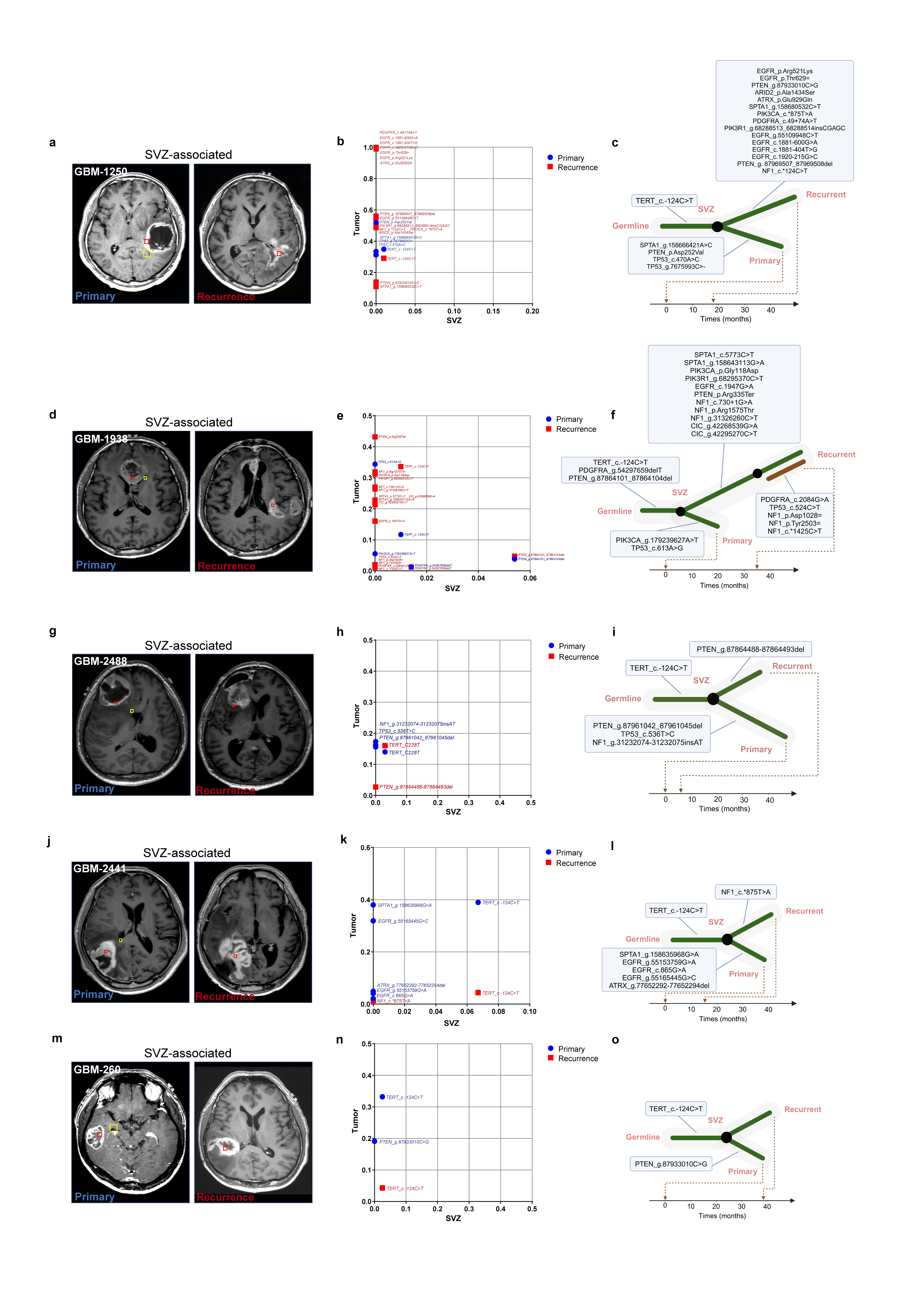
**

**Supplementary Fig. 2** SVZ NSCs evolve directly to recurrent glioblastoma.

**a-o** Pre-operation and recurrent brain MRI images, VAF scatterplots of mutations, and corresponding tumor phylogenetic evolutionary trees for each patient in the SVZ-associated recurrence group. Sampling sites are marked with boxes: red boxes indicate primary and recurrent tumors, and yellow boxes represent the SVZ.


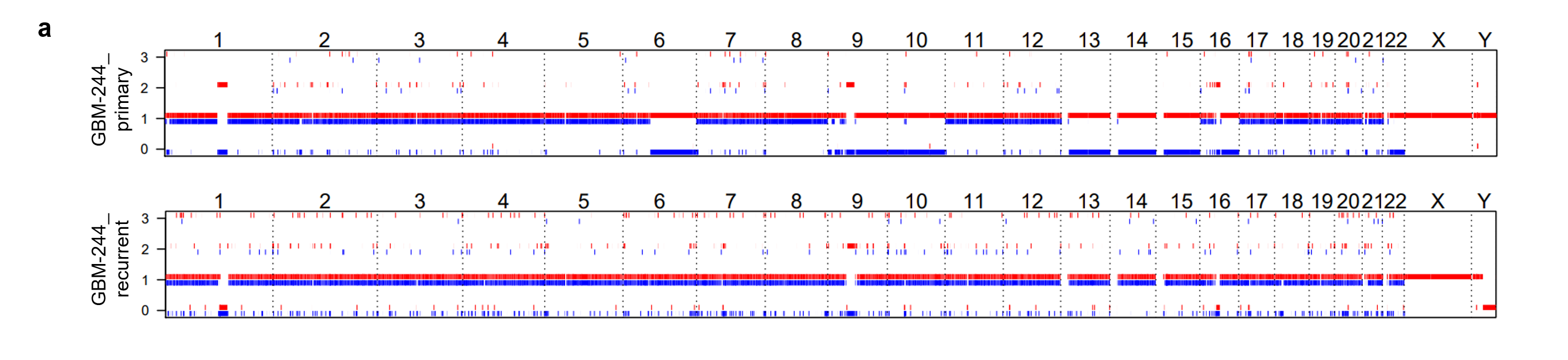


**Supplementary Fig. 3** Whole genome sequencing showing distinct copy number variation (CNV) patterns in longitudinal paired tumors.

Genome-wide CNV plots of primary and recurrent tumors from patient GBM-244.


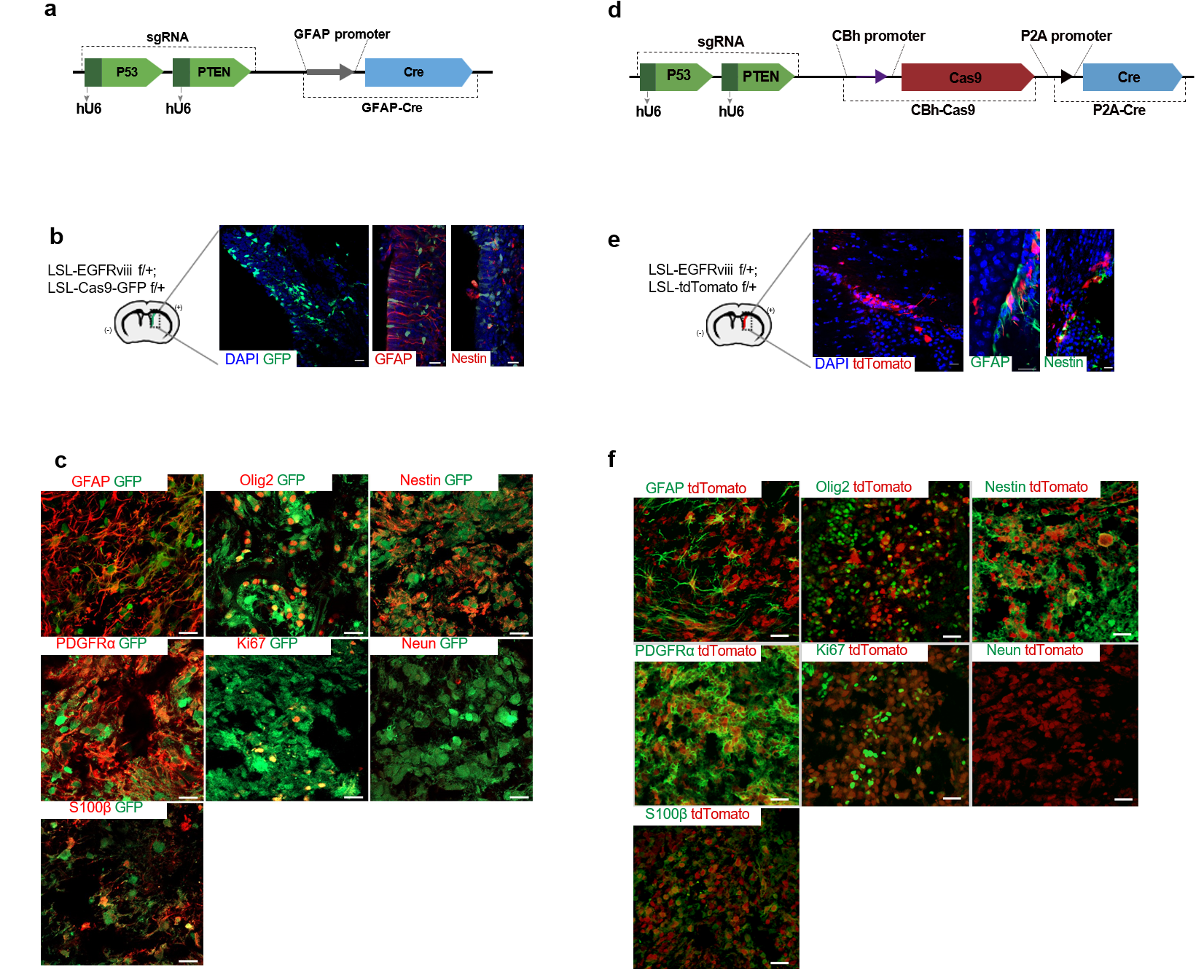


**Supplementary Fig. 4**  Reconstruction of high-grade glioma in genome-edited mice with cancer mutations in SVZ after surgical resection.

**a,** Map of a single vector with GFAP promoter and expressing Cre recombinase with the sgRNAs targeting *p53/Pten.*

**b,** Immunostaining image of GFP positive neural stem cells after electroporation of the vector in SVZ-mutated mice (left panel); the image highlights the localization of GFP-positive cells along the SVZ co-stained with GFA P and Nestin (middle and right panel). Scale bars, 20 µm.

**c,** Immunostaining of classic high-grade glioma-related markers in localized tumors of GFP positive localized tumors.

**d,** Map of a single vector expressing Cas9 and Cre recombinase with the sgRNAs targeting *p53/Pten.*

**e,** Immunostaining image of tdTomato positive neural stem cells after electroporation of the vector in SVZ-mutated mice (left panel); the image highlights the localization of tdTomato-positive cells along the SVZ co-stained with GFAP and Nestin (right panel). Scale bars, 20 µm.

**f,** Immunostaining of classic high-grade glioma-related markers in localized tumors, including nestin, GFAP, Olig2, S100β and Ki67, as well as the neuronal marker NeuN, in tumors (n=6). Scale bars, 20 μm.


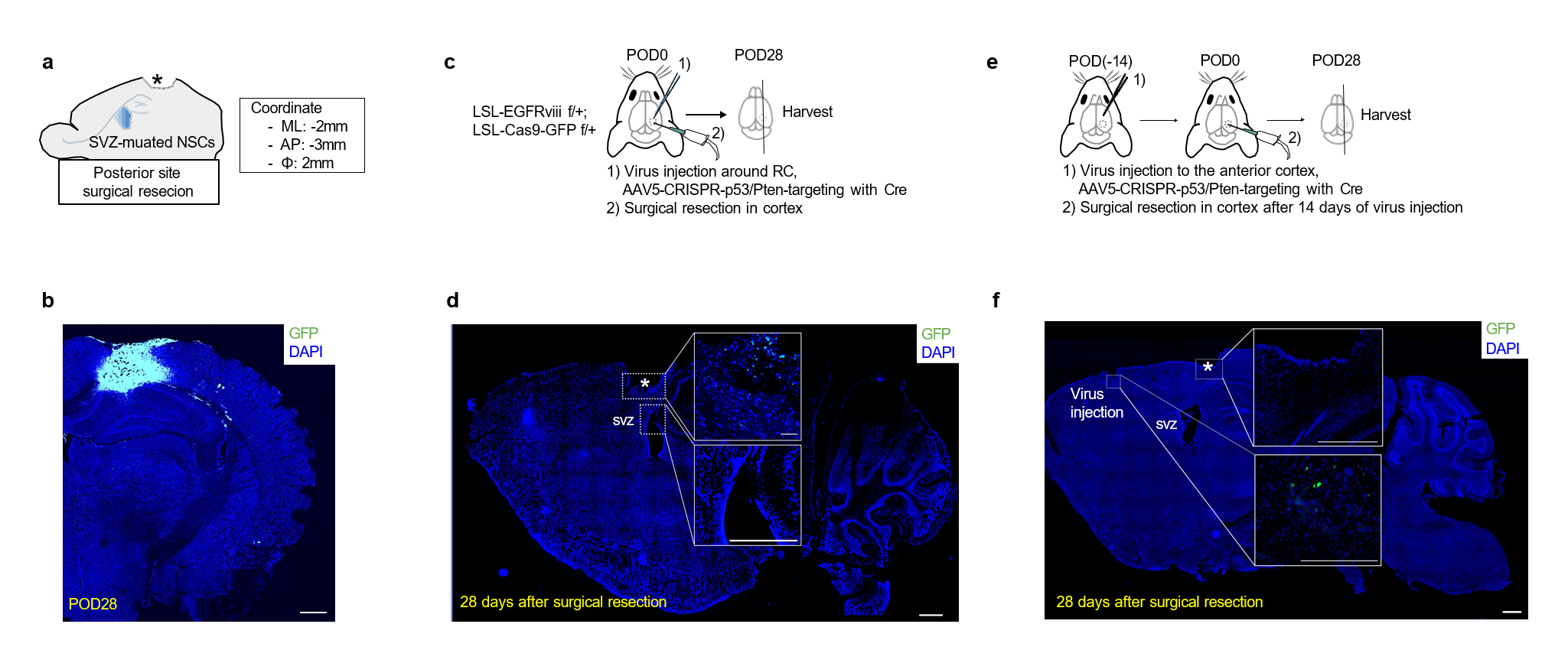
 **Supplementary Fig. 5** Mouse modeling of glioblastoma recurrence in genome-edited mice with cancer mutations in SVZ after surgical resection.

**a,** Illustration of the experimental procedure for surgical resection in posterior cortex.

**b,** Representative images showing local tumor developed in the resection site after 28 days of the procedure in panel g. Scale bars, 500 μm.

**c** Experimental scheme showing the procedure of viral injection of AAV5 containing sgRNAs for *P53* and *Pten* genes, with the expression of Cas9 and Cre recombinase into cortex, followed by surgical resection in 4 week-old LSL-EGFRviii;LSL-Cas9-GFP mice.

**d,** Representative images showing immigrating cell around the RC (upper panel, scale bar, 20 μm) and ipsilateral SVZ (lower panel, scale bar, 500 μm) after 28 days of the procedure in panel a. Scale bar, 500 μm.

**e,** Experimental scheme showing the procedure of the viral injection of AAV5 containing sgRNAs for *P53* and *Pten* genes, with the expression of Cas9 and Cre recombinase into cortex, followed by surgical resection after 2 weeks, in LSL-EGFRviii;LSL-Cas9-GFP mice.

**f,** Representative images showing immigrating cell around the RC (upper panel) and injection site (lower panel) after 28 days of the procedure in panel c. Scale bars, 500 μm.


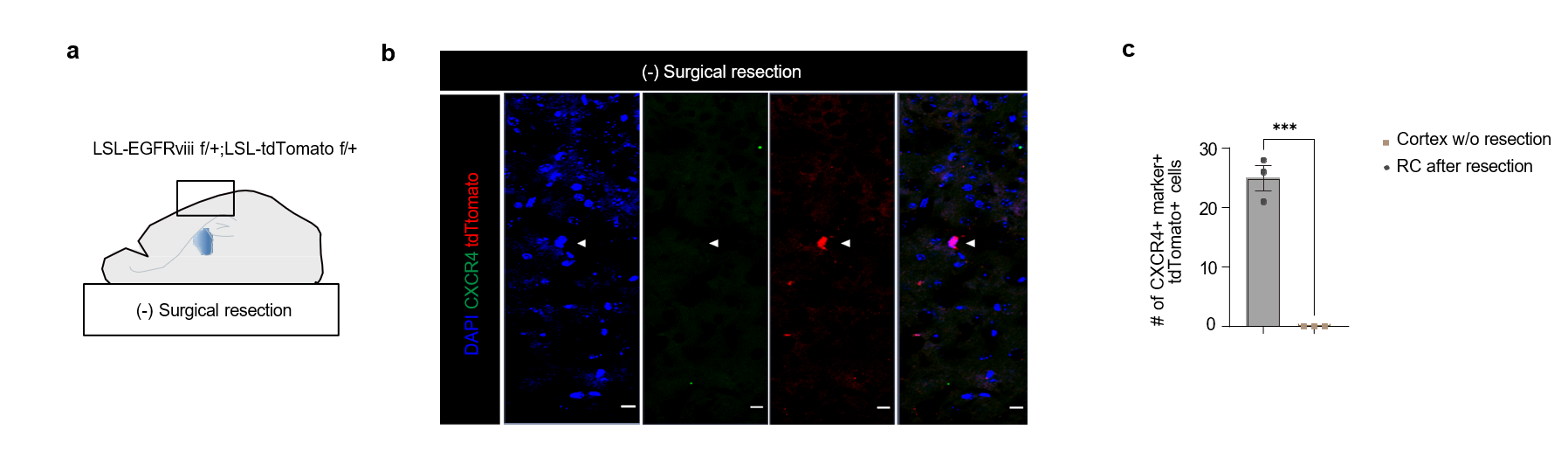


**Supplementary Fig. 6** Migration of NSCs in the absence of surgical resection is not driven by CXCL12-CXCR4 axis.

**a,** Illustration of the sampling site of normal cortex from mouse.

**b,** Representative immunostaining images of CXCR4 in the mouse normal cortex after 11 weeks without surgical resection. Scale bars, 50 µm, and the white arrowheads represent CXCR4-positive cells colocalizing with tdTomato+ cells. Scale bars, 50 µm.

**c,** Quantification of the number of CXCR4+ tdTomato+ cells around the RC on POD14 and in the normal cortex without surgical resection at 8-11 weeks. Statistical analysis was performed using a two-sided Student's t-test (***P < 0.001). Data are presented as mean ± SEM.

** Supplementary Fig. 7** Transcriptomic difference in recurrent tumors with distinct cellular origins.

**a**, Illustration of the strategy used for tumor sampling from transplanted tumor mouse model, involving dissection of the tumor tissues from the primary tdTomato+ tumor (P_TDT), recurrent tdTomato+ tumor (TDT), recurrent GFP+ tumor (GFP), and tumor constructed with a mixture of tdTomato+ and GFP+ cells. All of these tumor tissues were then subjected to RNA-sequencing.

**b,** Representative coronal sections of mice stained with DAPI showing sections from tumor-implanted mice after complete surgical removal of the primary tumor. The asterisk indicates the RC. The white dashed line indicates the SVZ and RC sites. Scale bars, 500 μm.

**c,** PCA analysis showing different gene expression distribution patterns in the tdTomato-positive primary tumor, tdToamto-positive recurrent tumor derived from residual tumor cells in RC, GFP-positive recurrent tumor derived from SVZ-mutated NSCs, and mixed tdTomato-positive and GFP-positive recurrent tumors derived from both residual tumor cells and SVZ-mutated NSCs.

**d,** Comparison of expression of CXCR4 and enrichment score of CXCR4 pathway between recurrent and primary tumors of mice. Statistical analysis was performed using a two-sided Student's t-test. Data are presented as mean ± SEM.


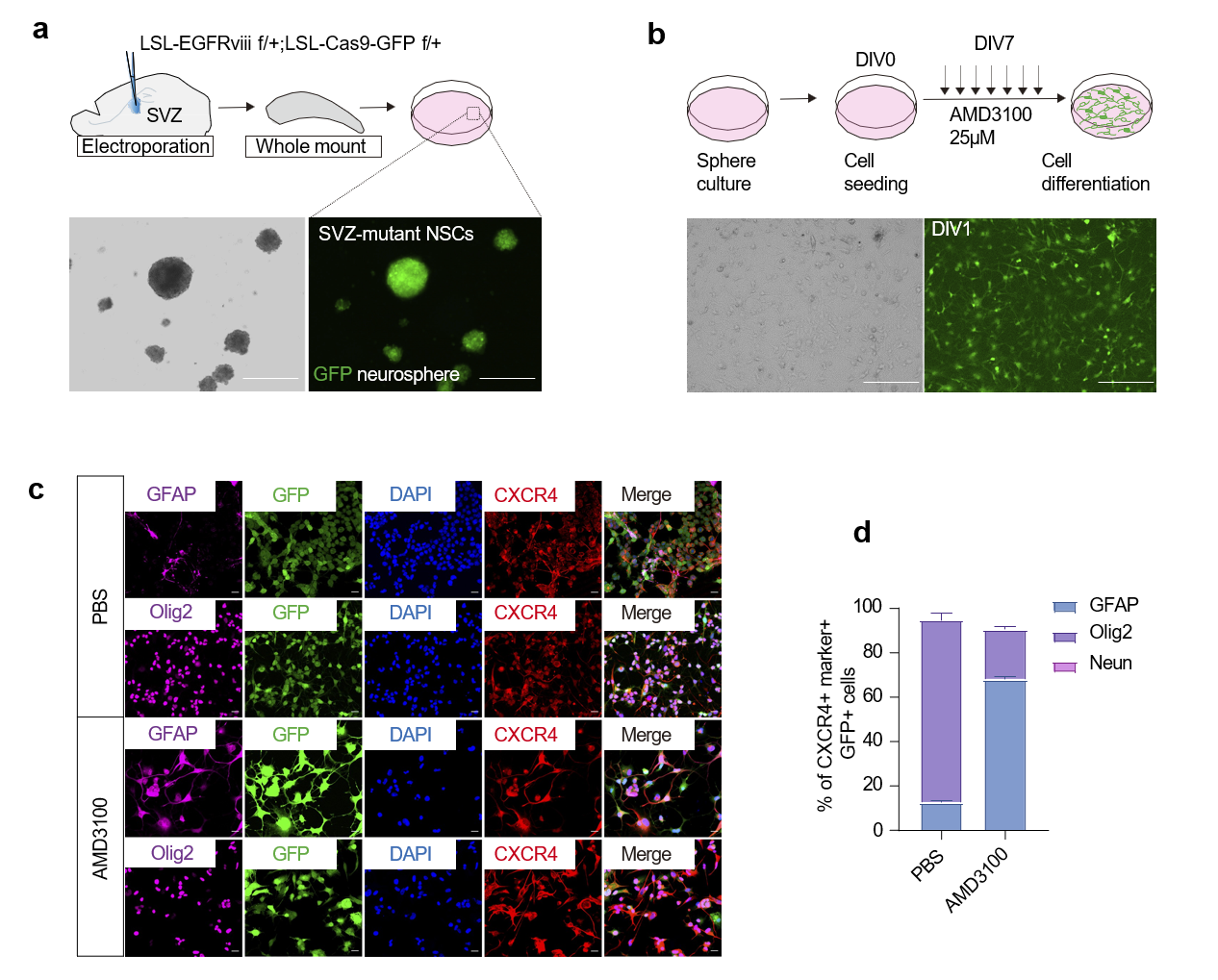
 **Supplementary Fig.8** Treatment via CXCL12/CXCR4 blockade decreased the number of OPC lineage cells in vitro.

**a&b,** Representative images of an undifferentiated tumorsphere and differentiatied monolayer cell growth after one day of incubation. SVZ-mutated cells were isolated and seeded in a confocal culture dish with triplicates. Scale bars, 200 µm.

**c,** Representative immunostaining images of GFAP, OLIG2 in cells 7 days after treatment with AMD3100, compared with the control group treated with PBS. Scale bars, 50 µm.

**d,** Quantification of the number of CXCR4+ cells that showed double-staining with GFAP, OLIG2, and NeuN in AMD3100 treatment, versus PBS group after seven days of differentiation.


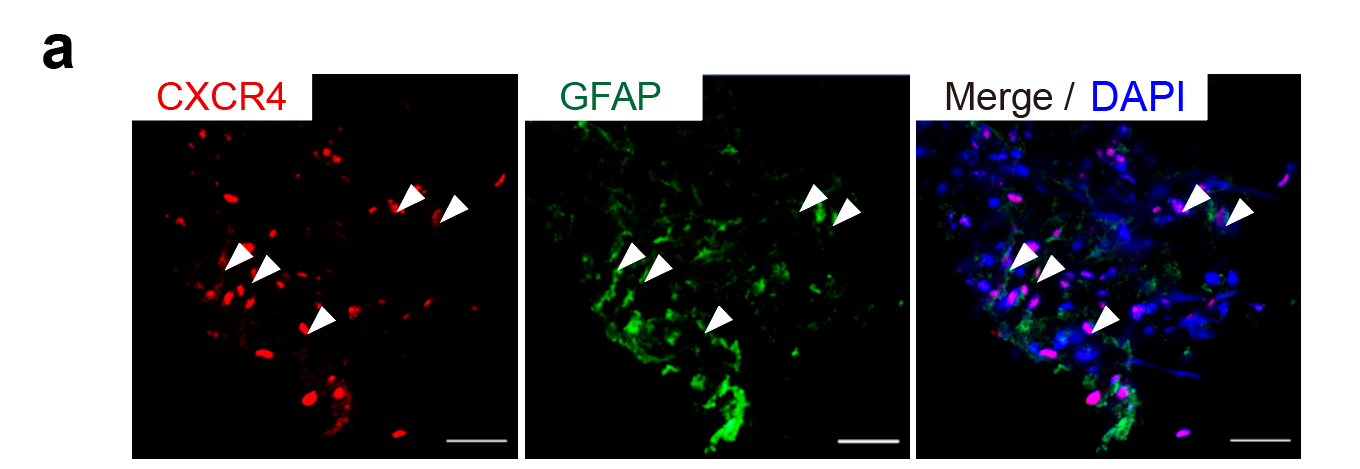


**Supplementary Fig. 9** Validation of CXCR4 expression in tumor cells and non-tumoral cells in local recurrent tumors in human patients with GBM.

**a,** Representative coronal sections of local recurrent tumors stained for GFAP (green) and CXCR4 (red). Co-localization of CXCR4 and GFAP, was indicated by white arrows. Scale bars, 50 µm.
